# Supplementary figures and images for: Deciphering chromatin architecture and dynamics in Plasmodium falciparum using the nucDetective pipeline
Source: PLoS Comput Biol. 2026 Jul 27;22(7):e1014557. doi: 10.1371/journal.pcbi.1014557 (PMC13426949; doi:10.1371/journal.pcbi.1014557)

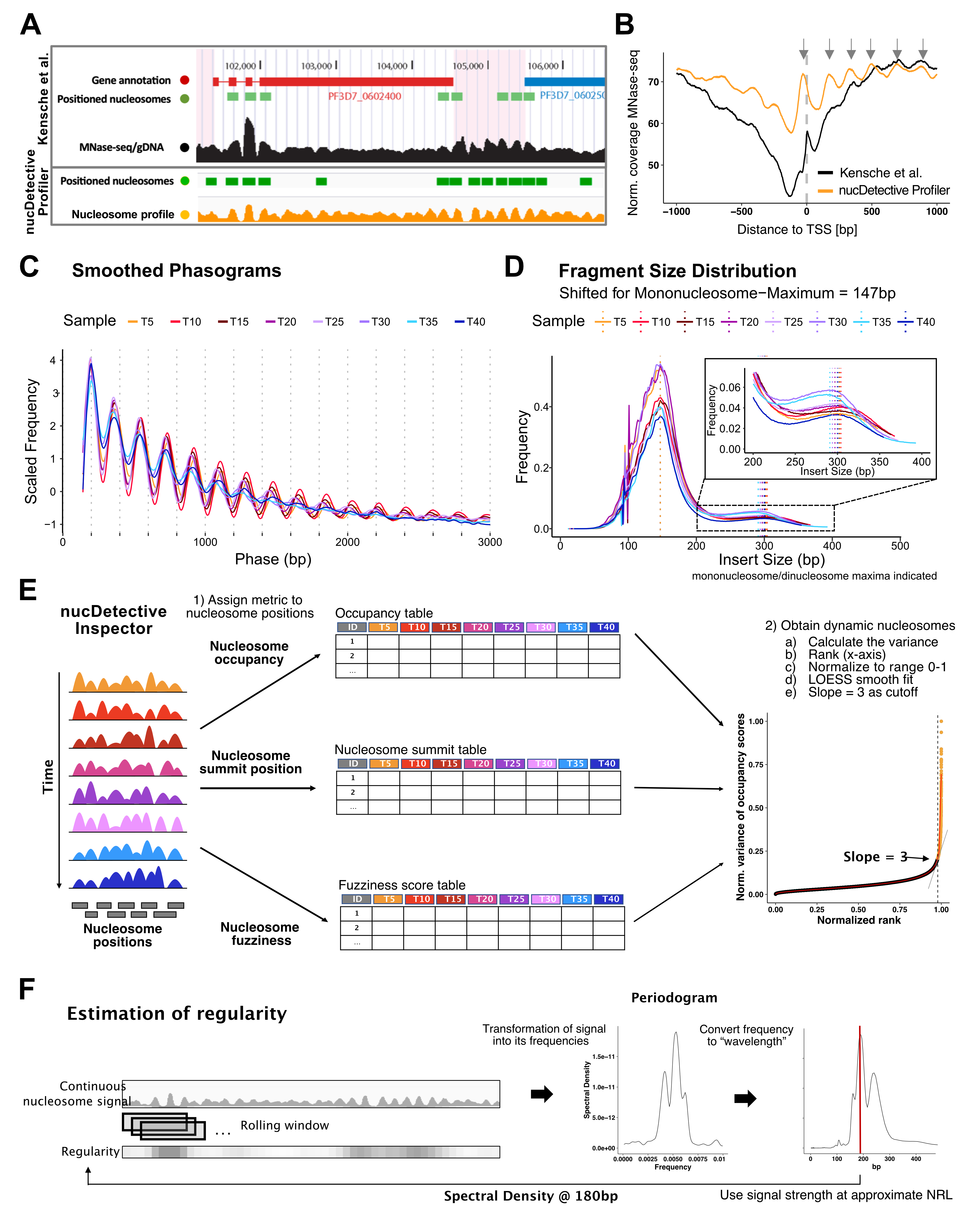

Supplement: S1 Fig — (A) The optimized MNase-seq data analysis workflow Profiler of the nucDetective pipeline improves the resolution of nucleosome positions in Pf. A comparison of detected positioned nucleosomes and nucleosome coverage at T5 is shown between the originally published analysis by Kensche and colleagues (top panel) [31] and our re-analysed data using the Profiler workflow of the nucDetective pipeline (bottom). This figure contains an edited figure from [31]. (B) Re-analyzed nucleosome TSS meta profile (yellow) exhibits phased nucleosomes (grey arrows) downstream of the TSS and a positioned +1 nucleosome located at the TSS. For comparison, the results of the original analysis by Kensche and colleagues (black) are shown [31]. (C) Smoothed phasograms from mononucleosomal DNA fragments at timepoints T5-T40. Phasograms were smoothed using LOESS regression (span = 0.03333), and frequency values were z-scaled to enable cross-timepoint comparison. (D) MNase-Seq fragment size distribution with the mononucleosome peak shifted to 147 bp to account for MNase digestion differences. Inset plot zooms into the dinucleosome peak (200 bp – 400 bp). Mono- and dinucleosome peaks are marked with dashed vertical lines. (E) Scheme outlining the Inspector workflow of the nucDetective pipeline to call nucleosomes with a change in occupancy, fuzziness or position shift over time. The analysis method of the different categories follows a common procedure: First, a score is assigned for each sample (here time point) to each nucleosome position. In case of position shifts, the exact dyad position at each timepoint is computed by loading the coverage track at the reference position, fitting a smooth curve and determining the summit position. In a second step, the resulting score matrix is used to calculate the variance for each nucleosome position over all time points. The resulting variance is normalized to a range between 0 and 1 (y-axis) and plotted against the ranks normalized by the total number [file pcbi.1014557.s001.tiff]

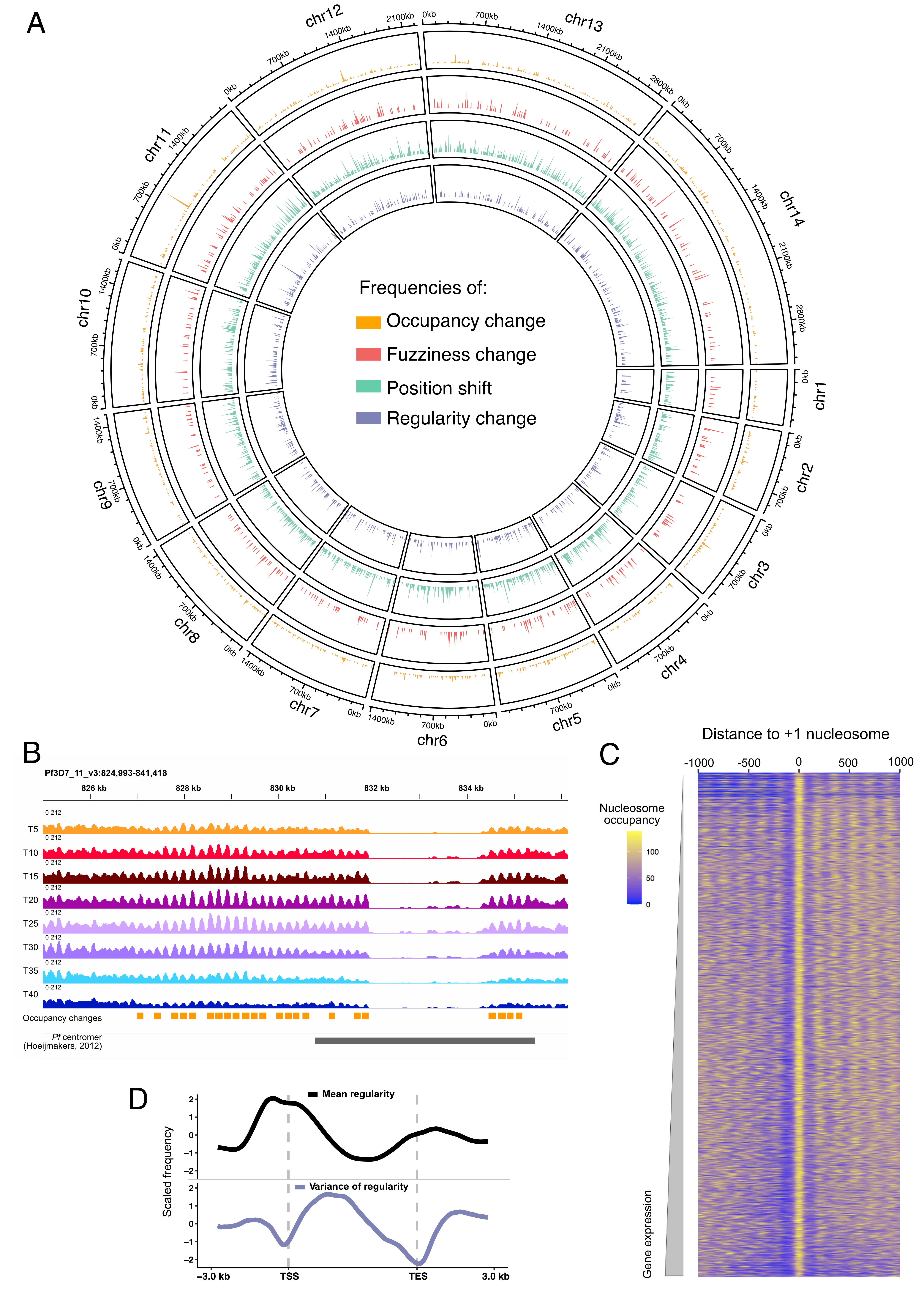

Supplement: S2 Fig — (A) Dynamic nucleosomes are evenly distributed across the entire genome on a global scale. Frequencies of nucleosomes showing occupancy (yellow), fuzziness (red), position (green) and regularity (blue) changes in 10 kb bins are depicted across the whole genome. (B) Genome browser snapshot illustrating accumulation of nucleosome occupancy changes at a centromeric site. Centered nucleosome coverage tracks (T5-T40 colored coverage tracks), nucleosomes occupancy changes (yellow bar) and annotated centromers (grey bar) taken from Hoeijmakers et al. [74]. (C) Nucleosome occupancy heatmap centered on the + 1 nucleosome, ordered by gene expression levels. Timepoint T20 is shown as an example. Occupancy values were winsorized at the 0.95 quantile to enhance visualization. Gene expression data represent rescaled RPKM values from Kensche et al.[31], obtained from GEO accession GSE66185. (D) Nucleosomes display regular spacing at the TSS, and changes of regularity during the IDC are primarily observed in the gene body. The meta profile of centered and scaled mean regularity (black) and variance of regularity (blue) is plotted over length scaled gene regions. Regularity is derived from the log10 spectral power at the period of 180 bp. TES = Transcription End Site. (TIFF) [file pcbi.1014557.s002.tiff]

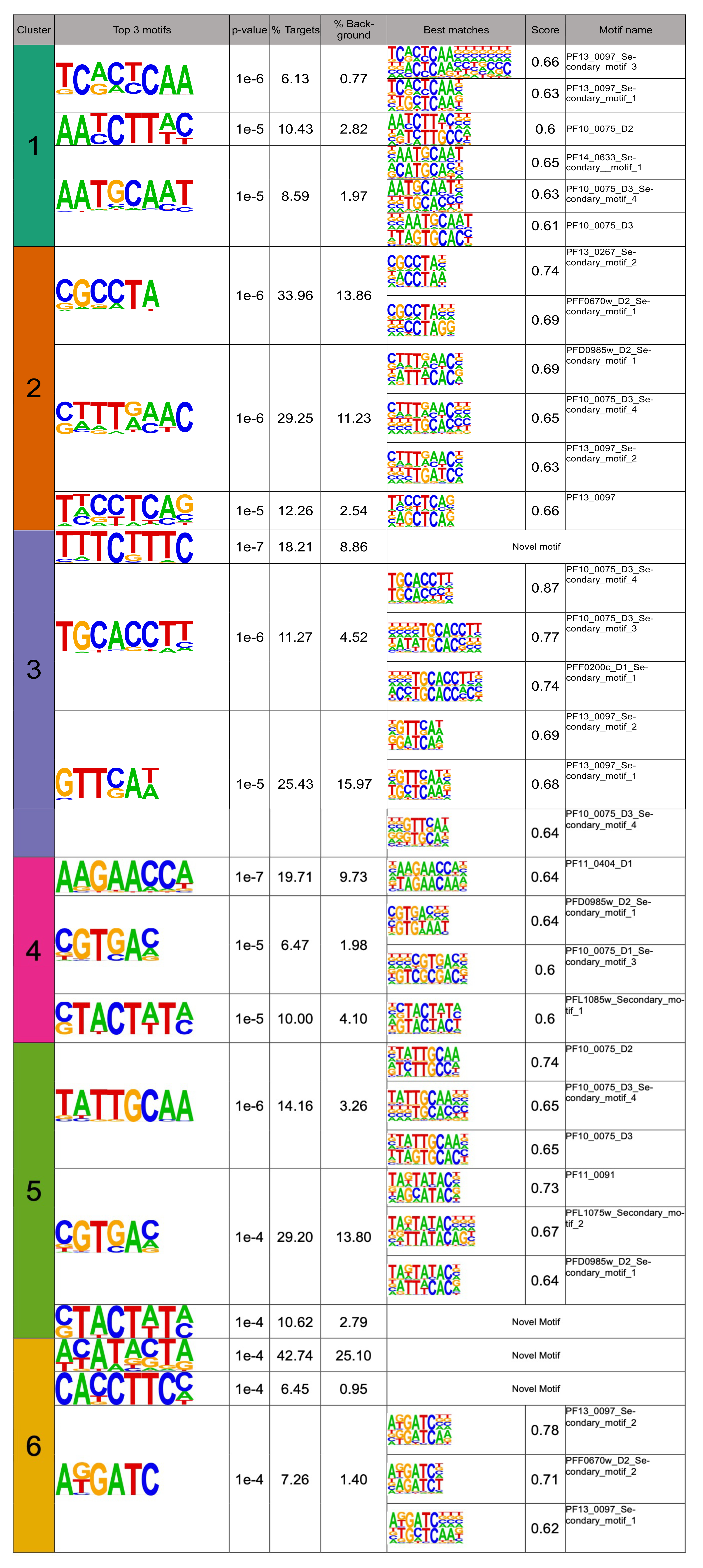

Supplement: S3 Fig — De novo DNA motifs enriched in distinct clusters of nucleosome occupancy changes (cf. clustering Fig 4A). Top 3 hits of each cluster are shown along with the significance of motif enrichment (hypergeometric test) and the fraction of motifs in dynamic nucleosome cluster or random background sequences. Known Pf transcription factor binding motifs taken from [45] with high similarity score (> 0.6) are shown next to it. (TIFF) [file pcbi.1014557.s003.tiff]

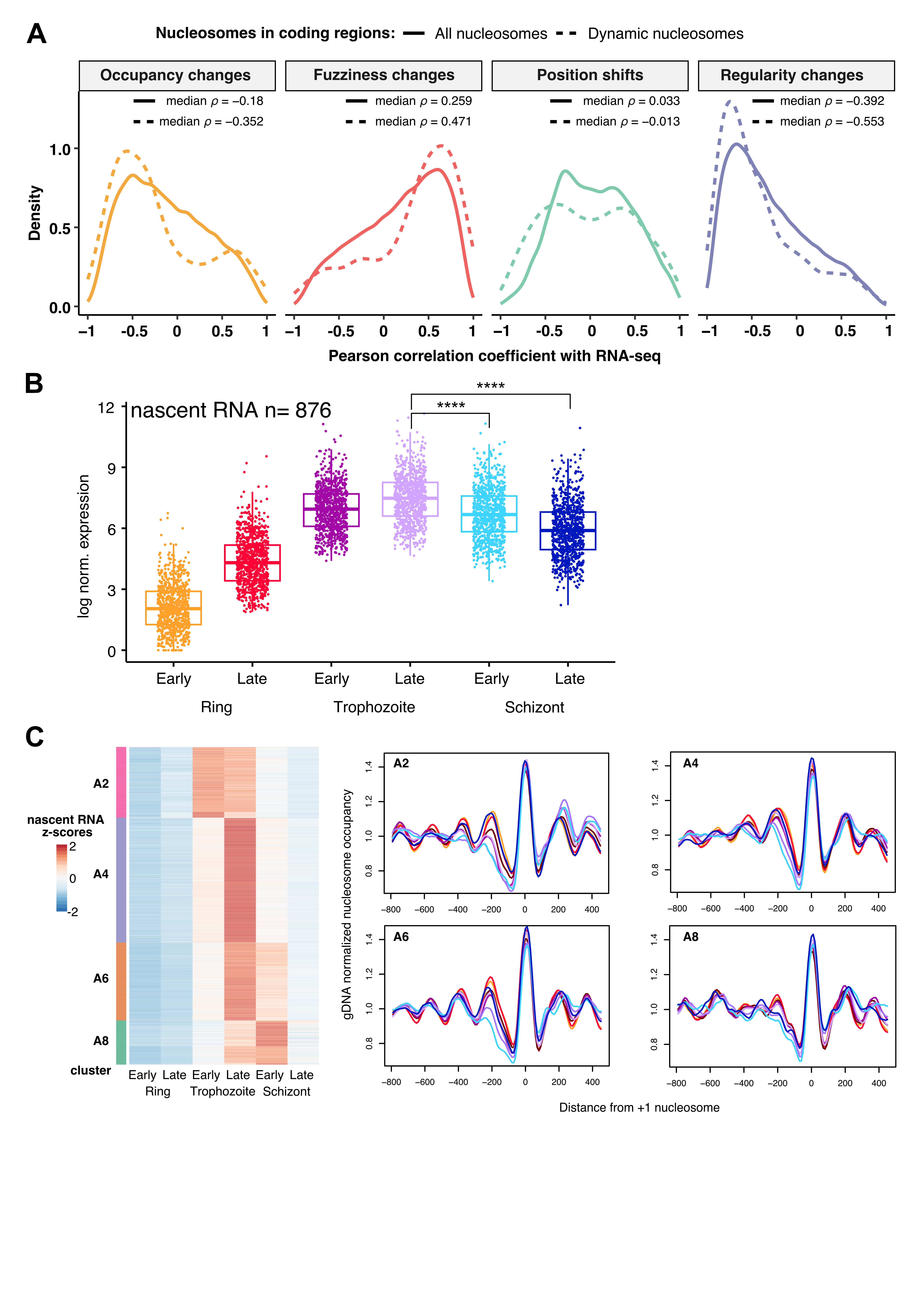

Supplement: S4 Fig — (A) Trends of nucleosome dynamics in coding regions during transcription. Linear correlations were computed for each nucleosome in coding regions, assessing the correlation between gene expression and occupancy, fuzziness, position shift and regularity over the Pf IDC. The density plot compares Pearson correlation coefficients for dynamic nucleosomes (dashed line) to those for all nucleosomes (solid line) in coding regions. The median Pearson correlation coefficient ρ for nucleosomes with high variance (dashed line) and for all nucleosomes (solid line) are indicated. (B) Normalized gene expression values obtained from GRO-seq data [48]. The same genes as shown in Fig 5A were taken. Paired t-test p ≤ 0.0001 (****). (C) Nucleosome features at the TSS of genes with distinct expression kinetics. Heatmap shows z-score scaled normalised nascent RNA levels measured by GRO-seq [48]. Spatio-temporal expression clustering of genes as indicated on the left side was taken from Lu and colleagues [48]. Nucleosome occupancy profiles centered at the + 1 nucleosomes of clustered genes show an opening of promoter region depending on transcriptional initiation (right). Nucleosome occupancy profiles were first scaled by the underlying profile of MNase digested gDNA and then the scaled coverage profile at each time point was divided by its region median coverage value. (TIFF) [file pcbi.1014557.s004.tiff]
