# Supplementary material for: Deciphering chromatin architecture and dynamics in Plasmodium falciparum using the nucDetective pipeline
Source: PLoS Comput Biol. 2026 Jul 27;22(7):e1014557. doi: 10.1371/journal.pcbi.1014557 (PMC13426949; doi:10.1371/journal.pcbi.1014557)
Supplement: Attachment — Submitted filename: 11-25 full-revision RC-2025-03175.pdf [file pcbi.1014557.s007.pdf]

Full Revision

Review COMMONS

Manuscript number: RC -2025-03175
Corresponding author(s): Gernot Längst

[Please use this template only if the submitted manuscript should be considered by the affiliate journal as a full revision in response to the points raised by the reviewers.

*If you wish to submit a preliminary revision with a revision plan, please use our "Revision Plan" template. **It is important to use the appropriate template to clearly inform the editors of your intentions.**]*

1. General Statements [optional]

This section is optional. Insert here any general statements you wish to make about the goal of the study or about the reviews.

2. Point-by-point description of the revisions

This section is mandatory. Please insert a point-by-point reply describing the revisions that were already carried out and included in the transferred manuscript.

Dear editor,
We have decided to return a full revision of the manuscript, addressing all points raised by the reviewers. This provides a detailed response to the reviewer comments, with our answers marked in blue color. In addition, we improved the manuscript accordingly and included additional Figures in the new version of the manuscript.
We thank the reviewers for their efforts and detailed evaluation of our manuscript. We think that the comments of the reviewers allowed us to significantly improve the manuscript.
With best regards
The authors of the manuscript

Reviewer #1 (Evidence, reproducibility and clarity (Required)):

Summary:
Holzinger et al. present a new automated pipeline, nucDetective, designed to provide accurate nucleosome positioning, fuzziness, and regularity from MNase-seq data. The pipeline is structured around two main workflows-Profiler and Inspector-and can also be applied to time-series datasets. To demonstrate its utility, the authors re-analyzed a Plasmodium falciparum MNase-seq time-series dataset (Kensche et al., 2016), aiming to show that nucDetective can reliably characterize nucleosomes in challenging AT-rich genomes. By integrating additional datasets (ATAC-seq, RNA-seq, ChIP-seq), they argue that the nucleosome positioning results from their pipeline have biological relevance.

Major Comments:

Despite being a useful pipeline, the authors draw conclusions directly from the pipeline's output without integrating necessary quality controls. Some claims either contradict existing literature or rely on misinterpretation or insufficient statistical support. In some instances, the pipeline output does not align with known aspects of Plasmodium biology.

I outline below the key concerns and suggested improvements to strengthen the manuscript and validate the pipeline:

• **Clarification of +1 Nucleosome Positioning in P. falciparum**

The authors should acknowledge that +1 nucleosomes have been previously reported in P. falciparum. For example, Kensche et al. (2016) used MNase-seq to map ~2,278 TSSs (based on enriched 5′-end RNA data) and found that the +1 nucleosome is positioned directly over the TSS in most genes: "Analysis of 2278 start sites uncovered positioning of a +1 nucleosome right over the TSS in almost all analysed regions" (Figure 3A).

They also described a nucleosome-depleted region (NDR) upstream of the TSS, which varies in size, while the +1 nucleosome frequently overlaps the TSS. The authors should nuance their claims accordingly. Nevertheless, I do agree that the +1 positioning in P. falciparum may be fuzzier as compared to yeast or mammals. Moreover, the correlation between +1 nucleosome occupancy and gene expression is often weak and that several genes show similar nucleosome profiles regardless of expression level. This raises my question: did the authors observe any of these patterns in their new data?

We appreciate the reviewer's insightful comment and agree that +1 nucleosomes and nucleosome depleted promoter regions have been previously reported in P. falciparum, notably by the Bartfai and Le Roch groups, including Kensche et al. (PMID: 26578577). Our study advances this understanding by providing, for the first time, a comprehensive view of the entirety of a canonical eukaryotic promoter architecture in P. falciparum—encompassing the NDR, the well-positioned +1 nucleosome, and the downstream phased nucleosome array. This downstream nucleosome array structure has not been characterized before, as prior studies noted a "lack of downstream nucleosomal arrays" (PMID: 26578577) or "relatively random" nucleosome organization within gene bodies (PMID: 24885191). We have revised the manuscript to more clearly acknowledge previous work and highlight our contributions. The changes we applied in the manuscript are highlighted in yellow and shown as well below.

In the Abstract L26-L230:

Contrary to the current view of irregular chromatin, we demonstrate for the first time regular phased nucleosome arrays downstream of TSSs, which, together with the established +1 nucleosome and upstream nucleosome-depleted region, reveal a complete canonical eukaryotic promoter architecture in *Pf*.

Introduction L156-L159:

For example, we identify a phased nucleosome array downstream of the TSS. Together with a well-positioned +1 nucleosome and an upstream nucleosome-free region. These findings support a promoter architecture in *Pf* that resembles classical eukaryotic promoters (Bunnik et al. 2014, Kensche et al. 2016).

Results L181-L183:

These new *Pf* nucleosome maps reveal a nucleosome organisation at transcription start sites (TSS) reminiscent of the general eukaryotic chromatin structure, featuring a reported well-positioned +1 nucleosome , an upstream nucleosome-free region (NFR, Bunnik et al. 2014, Kensche et al. 2016), and shown for the first time in *Pf*, a phased nucleosome array downstream of the TSS.

Discussion L414-L419:

Previous analyses of *Pf* chromatin have identified +1 nucleosomes and NFRs (Bunnik et al 2014, Kensche et al. 2016). Here we extend this understanding by demonstrating phased nucleosome array

structures throughout the genome. This finding provides evidence for a spatial regulation of nucleosome positioning in Pf, challenging the notion that nucleosome positioning is relatively random in gene bodies (Bunnik et al. 2014, Kensche et al. 2016). Consequently our results contribute to the understanding that Pf exhibits a typical eukaryotic chromatin structure, including well-defined nucleosome positioning at the TSS and regularly spaced nucleosome arrays (Schones et al. 2008; Yuan et al. 2005).
Regarding the reviewer's question on +1 nucleosome dynamics. Our data agrees with the reviewer and other studies (e.g. PMID: 31694866), that the +1 nucleosome position is robust and does not correlate with gene expression strength. In the manuscript we show that dynamic nucleosomes are preferentially detected at the –1 nucleosome position (Figure 2C). In line with that we show that the +1 nucleosome position does not markedly change during transcription initiation of a subset of late transcribed genes (Figure 5A). However, we observe an opening of the NDR and within the gene body increased fuzziness and decreased nucleosome array regularity (Figure S4A). To illustrate the relationship between the +1 nucleosome positioning and expression strength, we have included a heatmap showing nucleosome occupancy at the TSS, ordered according to expression strength (NEW Figure S4C):

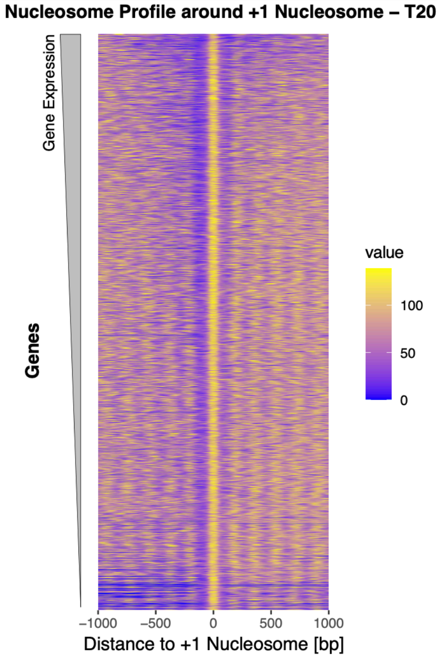

We included a sentence describing the relationship of +1 nucleosome position with gene expression in L257-L258:
Furthermore, the +1 nucleosome positioning is unaffected by the strength of gene expression (Figure S2C).

• Lack of Quality Control in the Pipeline
The authors claim (lines 152-153) that QC is performed at every stage, but this is not supported by the implementation. On the GitHub page (GitHub - uschwartz/nucDetective), QC steps are only marked at the Profiler stage using standard tools (FastQC, MultiQC). The Inspector stage, which is crucial for validating nucleosome detection, lacks QC entirely.
The authors should implement additional steps to assess the quality of nucleosome calls. For example, how are false positives managed? ROC curves should be used to evaluate true positive vs. false positive rates when defining dynamic nucleosomes. How sequencing biases are adressed?
The workflow overview chart on GitHub was not properly color coded. Therefore, we changed the graphics and highlighted the QC steps in the overview charts accordingly:

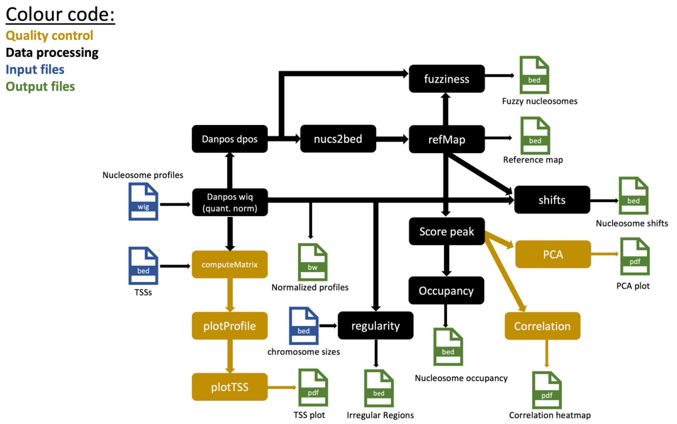

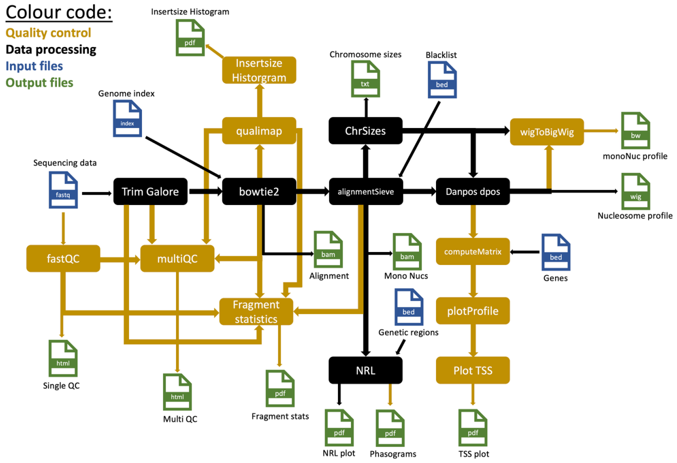

Based on our long standing expertise of analysing MNase-seq data (PMID: 38959309, PMID: 37641864, PMID: 30496478, PMID: 25608606), the best quality metrics to assess the performance of the challenging MNase experiment are the fragment size distributions revealing the typical nucleosomal DNA lengths and the TSS plots showing a positioned +1 nucleosome and regularly phased nucleosome arrays downstream of the +1 nucleosome. Additionally, visual inspection of the nucleosome profiles in a genome browser is advisable. We make those quality metrics easily available in the nucDetective Profiler workflow (Insertsize Histogram, TSS plot and provide nucleosome profile bigwig files). Furthermore, the PC and correlation analysis based on the nucleosome occupancy in the inspector workflow allows to evaluate replicate reproducibility or integrity of time series data, as shown for data evaluated in this manuscript.
The inspector workflow uses the well-established DANPOS toolkit to call nucleosome positions. Based on our experience, this step is particularly robust and well-established in the DANPOS toolkit (PMID: 23193179), so there is no need to reinvent it. Nevertheless, appropriate pre-processing of the data as done in the nucDetective pipeline is crucial to obtain highly resolved nucleosome positions. Using the final nucleosome profiles (bigwig) and the nucleosome reference positions (bed) as output of the Inspector workflow allows visual inspection of the called nucleosomes in a genome viewer. Furthermore, to avoid using false positive nucleosome positions for dynamic nucleosome analysis, we take only the 20% best positioned nucleosomes of each sample, as determined by the fuzziness score.
We understand the value of a gold standard of dynamic nucleosomes to test performance using ROC curves. However, we are not aware that such a gold standard exists in the nucleosome analysis field, especially not when using multi-sample settings, such as time series data. One alternative would be to use simulated data; however, this has several limitations:

- **Lack of biological complexity:** simulated data often fails to capture the full complexity of biological systems including the heterogeneity, variability, and subtle dependencies present in real-world data. Simplifications and omissions in simulation models can result in test datasets that are more tractable but less realistic, causing software to appear robust or accurate under idealized conditions, while underperforming on actual experimental data.
- **Risks of Overfitting:** Software may be tuned to perform well on simulated datasets leading to overfitting and falsely inflated performance metrics. This undermines the predictive or diagnostic value of the results for real biological data
- **Poor Model Fidelity and Hidden Assumptions:** The authenticity of simulated data is bounded by the fidelity of the underlying models. If those models are inaccurate or make untested assumptions, the generated data may not reflect real experimental or clinical scenarios. This can mask software shortcomings or bias validation toward specific, perhaps irrelevant, scenarios.

Therefore, we decided to validate the performance of the pipeline in the biological context of the analyzed data:

1) PCA analysis of the individual nucleosome features shows a cyclic structure as expected for the IDC (Fig. 1D-G).
2) Nucleosome occupancy changes anti-correlate with chromatin accessibility (Fig. 3B) as expected.
3) Dynamic nucleosome features correlate with expression changes (Fig. 5C)

We are aware that MNase-seq experiments might have sequence bias caused by the enzyme's endonuclease sequence preference (PMID: 30496478). However, the main aim of the nucDetective pipeline is to identify dynamic nucleosome features genome wide. Therefore, we are comparing the nucleosome features across multiple samples to find the positions in the genome with the highest variability. Comparisons are performed between the same nucleosome positions at the same genomic sites across multiple conditions, so the sequence context is constant and does not confound the analysis. This is like the differential expression analysis of RNA-seq data, where the gene counts are not normalized by gene length. Introducing a sequence normalization step might distort and bias the results of dynamic nucleosomes.

We included a paragraph describing the limitations to the discussion (L447-457):
Depending on the degree of MNase digestion, preferentially nucleosomes from GC rich regions are revealed in MNase-seq experiments (Schwartz et al. 2019). However, no sequence or gDNA normalisation step was included in the nucDetective pipeline. To identify dynamic nucleosomes, comparisons are performed between the same nucleosome positions at the same genomic sites across multiple samples. Hence, the sequence context is constant and does not confound the analysis. Introducing a sequence normalization step might even distort and bias the results. Nevertheless, it is highly advisable to use low MNase concentrations in chromatin digestions to reduce the sequence bias in nucleosome extractions. This turned out to be a crucial condition to obtain a homogenous nucleosome distribution in the AT-rich intergenic regions of eukaryotic genomes and especially in the AT-rich genome of *Pf* (Schwartz et al. 2019, Kensche et al. 2016).

• Use of Mono-nucleosomes Only
The authors re-analyze the Kensche et al. (2016) dataset using only mono-nucleosomes and claim improved nucleosome profiles, including identification of tandem arrays previously unreported in P. falciparum. Two key issues arise:

Review COMMONS

1. Is the apparent improvement due simply to focusing on mono-nucleosomes (as implied in lines 342-346)?

The default setting in nucDetective is to use fragment sizes of 140 – 200 bp, which corresponds to the main mono-nucleosome fraction in standard MNase-seq experiments. However, the correct selection of fragment sizes may vary depending on the organism and the variations in MNase-seq protocols. Therefore, the pipeline offers the option of changing the cutoff parameter (--minLen; --maxLen), accordingly. Kensche et al thoroughly tested and established the best parameters for the data set. We agree with their selected parameters and used the same cutoffs (75-175 bp) in this manuscript. For this particular data set, the fragment size selection is not the reason why we obtain a better resolution. MNase-seq analysis is a multistep process which is optimized in the nucDetective pipeline. Differences in the analysis to Kensche et al are at the pre-processing stage and alignment step:
Kensche et al. : "Paired-end reads were clipped to 72 bp and all data was mapped with BWA sample (Version 0.6.2-r126)"

nucDetective:

1) Trimming using TrimGalore --paired -q 10 --stringency 2
2) Mapping using bowtie2 --very-sensitive –dovetail --no-discordant
3) MAPQ >= 20 filtering of aligned read-pairs (samtools).

The manuscript text L379 was changed to

This is achieved using MNase-seq optimized alignment settings, and proper selection of the fragment sizes corresponding to mono-nucleosomal DNA to obtain high resolution nucleosome profiles.

2. How does the pipeline perform with di- or tri-nucleosomes, which are also biologically relevant (Kensche et al., 2016 and others)?
Furthermore, the limitation to mono-nucleosomes is only mentioned in the methods, not in the results or discussion, which could mislead readers.

The pipeline is optimized for mono-nucleosome analysis. However, the cutoffs for fragment size selection can be adjusted to analyse other fragment populations in MNase-seq data (--minLen; --maxLen). For example we know from previous studies that the settings in the pipeline could be used for sub-nucleosome analysis as well (PMID: 38959309). Di- or Tri-nucleosome analysis we have not explicitly tested. However, in a previous study (PMID: 30496478) we observed that the inherited MNase sequence bias is more pronounced in di-nucleosomes, which are preferentially isolated from GC-rich regions. This is in line with the depletion of di-nucleosomes in AT-rich intergenic regions in Pf, as was already described by Kensche et al.
Changes to the manuscript text: We included a paragraph describing the limitations to the discussion (L428-434):
The nucDetective pipeline has been optimized for the analysis of mono-nucleosomes. However, the selection of fragment sizes can be adjusted manually, enabling the pipeline to be used for other nucleosome categories. The pipeline is suitable to map and annotate sub-nucleosomal particles (< 140 bp) as well, however additional steps like histone immunoprecipitation and MNase titrations might be required to validate the sub-nucleosome structures (Wernig-Zorc et al. 2024). How the pipeline performs with fragments derived from multi-nucleosome structures like di- and tri-nucleosomes needs to be tested.

• Reference Nucleosome Numbers

The authors identify 49,999 reference nucleosome positions. How does this compare to previous analyses of similar datasets? This should be explicitly addressed.

We thank the reviewer for this suggestion. In order to put our results in perspective, it is important to distinguish between reference nucleosome positions (what we reported in the manuscript) and all detectable nucleosomes. The reference positions are our attempt to build a set of nucleosome positions with strong evidence, allowing confident further analysis across timepoints. The selection of a well positioned subset of nucleosomes for downstream analysis has been done previously (PMID: 26578577) and the merging algorithm we used across timepoints is also used by DANPOS to decide if a MNase-Seq peak is a new nucleosome position or belongs to an existing position (PMID: 23193179).

To be able to address the reviewer suggestion we prepared and added a table to the supplementary data, including the total number of all nucleosomes detected by our pipeline at each timepoint. We adjusted the results to the following (L223-226):

"The pipeline identified a total of 127370 ± 1151 (mean ± SD) nucleosomes at each timepoint (Supplementary Data X). To exclude false positive positions in our analysis, we conservatively selected 49,999 reference nucleosome positions, representing sites with a well-positioned nucleosome at least at one time point (see Methods). Among these 1192 nucleosomes exhibited […]"

Several groups reported nucleosome positioning data for *P. falciparum* (PMID: 20015349, PMID: 20054063, PMID: 24885191, PMID: 26578577), however only Ponts et al (2010) reported resolved numbers (~45000-90000 nucleosomes depending in development stage) and Bunnik et al reported ~ 75000 nucleosomes in a graph. Although we do not know the reason of why the other studies did not include specific numbers, we speculate that the data quality did not allow them to confidently report a number. In fact, nucleosomal reads are severely depleted in AT-rich intergenic regions in the Ponts and Bunnik datasets. In contrast, Kensche et al (and our analysis) shows that nucleosomes can be identified throughout the genome of *Pf*. Therefore, the nucleosome numbers reported by Ponts et al and Bunnik et al are very likely underestimated.

We included the following text in the discussion, addressing previously published datasets (L404 – 405):

"For example, our pipeline was able to identify a total of ~127,000 nucleosomes per timepoint (=5.4 per kb) in range with observed nucleosome densities in other eukaryotes (typically 5 to 6 per kb). From these, we extracted 49,999 reference nucleosome positions with strong positioning evidence across all timepoints, which we used to characterize nucleosome dynamics of *Pf* longitudinally. Previous studies of *P. falciparum* chromatin organization, did not report a total number of nucleosomes (Westenberger et al. 2009, Kensche et al. 2016), or estimated approximately ~45000-90000 nucleosomes across the genome at different developmental stages (Bunnik et al. 2014, Ponts et al. 2010). However, this value likely represents an underestimation due to the depletion of nucleosomal reads in AT-rich intergenic regions observed in their datasets."

• Figure 1B and Nucleosome Spacing

The authors claim that Figure 1B shows developmental stage-specific variation in nucleosome spacing. However, only T35 shows a visible upstream change at position 0. In A4, A6, and A8 (Figure S4), no major change is apparent. Statistical tests are needed to validate whether the observed differences are significant and should be described in the figure legends and main text.

We would like to thank the reviewer for bringing this issue to our attention. We apologize for an error we made, wrongly labelling the figure numbers. The differences in nucleosome spacing across time are visible in Figure 1C. Figure 1B shows the precise array structure of the *Pf* nucleosomes, when centered on the +1 nucleosome, and is mentioned before. The mistake is now corrected.

In Figure 1C the mean NRL and 95% confidence interval are depicted, allowing a visual assessment of data significance (non-overlapping 95% CI-Intervals correspond to $p < 0.05$). However, we share the reviewer's opinion that a formal statistical test would be ideal to confirm general differences. Unfortunately, to our knowledge, the way in which the NRLs are determined does not allow for a simple statistical test. NRLs are derived from a linear regression of the peak maxima of the accumulated 5' read distances, resulting in an average NRL and 95% confidence intervals for each sample. No ANOVA/Kruskal Wallis can be calculated from only mean and CI values. However, in order to be able to make a statistical estimate nonetheless, we used a bootstrapping-based approach, assuming normal distribution of NRLs and simulating 3 values per timepoint.
Taken together we corrected this mistake and edited the text as follows (L194 – 199):
"With this +1 nucleosome annotation, regularly spaced nucleosome arrays downstream of the TSS were detected, revealing a precise nucleosome organization in *Pf* (Figure 1B). Due to the high resolution maps of nucleosomes we can now observe significant variations in nucleosome spacing depending on the developmental stage (Figure 1C, ANOVA on bootstrapped values (3 per timepoint) $F_{7,72} = 35.10$, $p < 0.001$, generalized $\eta^2 = 0.773$)."

• Genome-wide Occupancy Claims

The claim that nucleosomes are "evenly distributed throughout the genome" (Figure S2A) is questionable. Chromosomes 3 and 11 show strong peaks mid-chromosome, and chromosome 14 shows little to no signal at the ends. This should be discussed.
Subtelomeric regions, such as those containing var genes, are known to have unique chromatin features. For instance, Lopez-Rubio et al. (2009) show that subtelomeric regions are enriched for H3K9me3 and HP1, correlating with gene silencing. Should these regions not display different nucleosome distributions? Do you expect the Plasmodium genome (or any genome) to have uniform nucleosome distribution?
On global scale (> 10 kb) we would expect a homogenous distribution of nucleosomes genome wide, regardless of euchromatin or heterochromatin. We have shown this in a previous study for human cells (PMID: 30496478), which was later confirmed for drosophila melongaster (PMID: 31519205,PMID: 30496478) and yeast (PMID: 39587299).
However, Figure S2A shows the distribution of the dynamic nucleosome features during the IDC, called with our pipeline. We agree with the reviewer, that there are a few exceptions of the uniform distribution, which we address now in the manuscript.
Furthermore, we agree with the reviewer that the H3K9me3 / HP1 subtelomeric regions are special. Those regions are depleted of dynamic nucleosomes in the IDC as shown in Fig. 2D and now mentioned in L280 - L282.
We included an additional genome browser snapshot in Supplemental Figure S2B and changed the text accordingly (L245-249):
We observed a few exceptions to the even distribution of the nucleosomes in the center of chromosome 3, 11 and 12, where nucleosome occupancy changes accumulated at centromeric regions (Figure S2B). Furthermore, the ends of the chromosomes are rather depleted of dynamic nucleosome features.

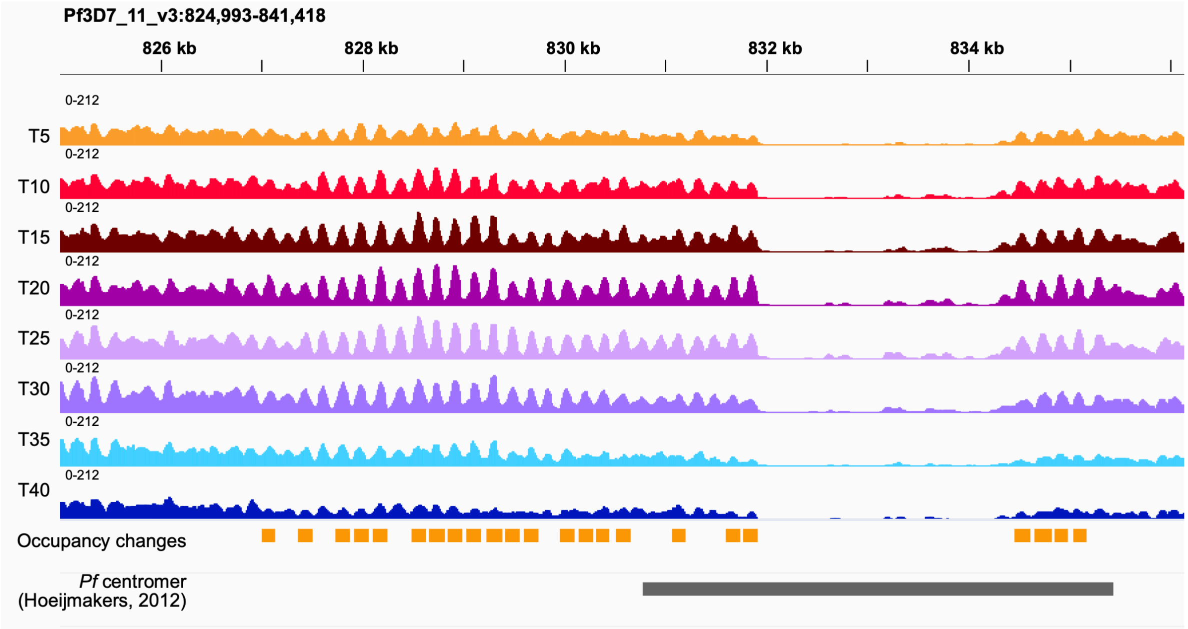

Genome browser snapshot illustrating accumulation of nucleosome occupancy changes at a centromeric site. Centered nucleosome coverage tracks (T5-T40 colored coverage tracks), nucleosomes occupancy changes (yellow bar) and annotated centromers (grey bar) taken from (Hoeijmakers et al., 2012)

• Dependence on DANPOS

The authors criticize the DANPOS pipeline for its limitations but use it extensively within nucDetective. This contradiction confuses the reader. Is nucDetective an original pipeline, or a wrapper built on existing tools?

One unique feature of the nucDetective pipeline is to identify dynamic nucleosomes (occupancy, fuzziness, regularity, shifts) in complex experimental designs, such as time series data (Inspector workflow). To our knowledge, there is no other tool for MNase-seq data which allows multi-condition/time-series comparisons (PMID: 35061087). For example, DANPOS allows only pair-wise comparisons, which cannot be used for time-series data. For the analysis of dynamic nucleosome features we require nucleosome profiles and positions at high resolution. For this purpose, several tools do already exist (PMID: 35061087). However, researchers without experience in MNase-seq analysis often find the plethora of available tools overwhelming, which makes it challenging to select the most appropriate ones. Here we share our experience and provide the user an automated workflow (Profiler), which builds on existing tools.

In summary the Profiler workflow is a wrapper built on existing tools and the Inspector workflow is partly a wrapper (uses DANPOS to normalize nucleosome profiles and call nucleosome positions) and implements our original algorithm to detect dynamic nucleosome features in multiple conditions / time-series data.

• Control Data Usage

The authors should clarify whether gDNA controls were used throughout the analysis, as done in Kensche et al. (2016). Currently, this is mentioned only in the figure legend for Figure 5, not in the methods or results.

Review COMMONS

We used the gDNA normalisation to optimize the visualization of the nucleosome depleted region upstream of the TSS in Fig 5A. Otherwise, we did not normalize the data by the gDNA control. The reason is the same as we did not include sequence normalization in the pipeline (see comment above)
We included a paragraph describing the limitations to the discussion (L447-457):
Depending on the degree of MNase digestion, preferentially nucleosomes from GC rich regions are revealed in MNase-seq experiments (Schwartz et al. 2019). However, no sequence or gDNA normalisation step was included in the nucDetective pipeline. To identify dynamic nucleosomes, comparisons are performed between the same nucleosome positions at the same genomic sites across multiple samples. Hence, the sequence context is constant and does not confound the analysis. Introducing a sequence normalization step might even distort and bias the results. Nevertheless, it is highly advisable to use low MNase concentrations in chromatin digestions to reduce the sequence bias in nucleosome extractions. This turned out to be a crucial condition to obtain a homogenous nucleosome distribution in the AT-rich intergenic regions of eukaryotic genomes and especially in the AT-rich genome of *Pf* (Schwartz et al. 2019, Kensche et al. 2016).
We added following statement to the methods part:
Additionally, the TSS profile shown in Figure 5A was normalized by the gDNA control for better NDR visualization.

• Lack of Statistical Power for Time-Series Analyses
Although the pipeline is presented as suitable for time-series data, it lacks statistical tools to determine whether differences in nucleosome positioning or fuzziness are significant across conditions. Visual interpretation alone is insufficient. Statistical support is essential for any differential analysis.
We understand the value of statistical support in such an analysis. However, in biology we often face the limitations in terms of the appropriate sample sizes needed to accurately estimate the variance parameters required for statistical modeling. As MNase-seq experiments require a large amount of input material and high sequencing depth, the number of samples in most experiments is low, often with only two replicates (PMID: 23193179). Therefore, we decided that the nucDetective pipeline should be rather handled as a screening method to identify nucleosome features with high variance across all conditions. This prevents misuse of p-values. A common misinterpretation we observed is the use of non-significant p-values to conclude that no biological change exists, despite inadequate statistical power to detect such changes. We included a paragraph in the limitations section discussing the limitations of statistical analysis of MNase-Seq data.
Changes to the manuscript text:
We included a paragraph describing the limitations to the discussion (L435-446).
As MNase-seq experiments require a large amount of input material and high sequencing depths, most published MNase-seq experiments do not provide the appropriate sample sizes required to accurately estimate the variance parameters necessary for statistical modelling (Chen et al. 2013). Therefore, dynamic nucleosomes are not identified through statistical testing but rather by ranking nucleosome features according to their variance across all samples and applying a variance threshold to distinguish them. This concept is well established to identify super-enhancers (Whyte et al. 2013). In this study we set the variance cutoff to a slope of 3, resulting in a high data confidence. However, other data sets might require further adjustment of the variance cutoff, depending on data quality or sequencing depth. The nucDetective identification of dynamic nucleosomes can be seen as a screening approach to provide a holistic overview of nucleosome dynamics in the system, which provides a basis for further research.

• **Reproducibility of Methods**
The Methods section is not sufficient to reproduce the results. The GitHub repository lacks the necessary code to generate the paper's figures and focuses on an exemplary yeast dataset. The authors should either:
o Update the repository with relevant scripts and examples,
o Clearly state the repository's purpose, or
o Remove the link entirely.
Readers must understand that nucDetective is dedicated to assessing nucleosome fuzziness, occupancy, shift, and regularity dynamics-not downstream analyses presented in the paper.

We thank the reviewer for this helpful comment. In addition to the main nucDetective repository, a second GitHub link is provided in the Data Availability section, which contains the scripts used to generate the figures presented in the paper. This separation was intentional to distinguish the general-purpose nucDetective tool from the project-specific analyses performed for this study. We acknowledge that this may not have been sufficiently clear.
To have all resources available at a single citable permanent location we included a link to the corresponding Zenodo repository (https://doi.org/10.5281/zenodo.16779899) in the Data and materials availability statement.
The Zenodo repository contains:
Code (scripts.zip) and annotation of Plasmodium falciparum (Annotation.zip) to reproduce the nucDetective v1.1 (nucDetective-1.1.zip) analysis as done in the research manuscript entitled "Deciphering chromatin architecture and dynamics in Plasmodium falciparum using the nucDetective pipeline".
The folder "output_nucDetective" conains the complete output of the nucDetective analysis pipeline as generated by the "01_nucDetective_profiler.sh" and "02_nucDetective_inspector.sh" scripts.
Nucleosome coverage tracks, annotation of nucleosome positions and dynamic nucleosomes are deposited additonally in the folder "Pf_nucleosome_annotation_of_nucDetective".
To make this clearer we added following text to Material and Methods in ”The nucDetective pipeline” section:
Changes in the manuscript text (L518-519):
The code, software and annotations used to run the nucDetective pipeline along with the output have been deposited on Zenodo (https://doi.org/10.5281/zenodo.16779899).

• **Supplementary Tables**
Including supplementary tables showing pipeline outputs (e.g., nucleosome scores, heatmaps, TSS extraction) would help readers understand the input-output structure and support figure interpretations.
See comments above.
We included a link to the corresponding Zenodo repository (https://doi.org/10.5281/zenodo.16779899) in the Data and materials availability statement.
The repository contains:
Code (scripts.zip) and annotation of Plasmodium falciparum (Annotation.zip) to reproduce the nucDetective v1.1 (nucDetective-1.1.zip) analysis as done in the research manuscript entitled "Deciphering chromatin architecture and dynamics in Plasmodium falciparum using the nucDetective pipeline".

Full Revision

The folder "output_nucDetective" conains the complete output of the nucDetective analysis pipeline as generated by the "01_nucDetective_profiler.sh" and "02_nucDetective_inspector.sh" scripts.

Minor Comments:

• The authors should moderate claims such as "no studies have reported a well-positioned +1 nucleosome" in P. falciparum, as this contradicts existing literature. Similarly, avoid statements like "poorly understood chromatin architecture of Pf," which undervalue extensive prior work (e.g., discovery of histone lactylation in Plasmodium, Merrick et al., 2023).

We would like to clarify that we neither wrote that ""no studies have reported a well-positioned +1 nucleosome"" in *P. falciparum* nor did we intend to imply such thing. However, we acknowledge that our original wording may have been unclear. To address this, we have revised the manuscript to explicitly acknowledge prior studies on chromatin organization and highlight our contribution.

In the Abstract L26-L30:

Contrary to the current view of irregular chromatin, we demonstrate for the first time regular phased nucleosome arrays downstream of TSSs, which, together with the established +1 nucleosome and upstream nucleosome-depleted region, reveal a complete canonical eukaryotic promoter architecture in *Pf*.

Introduction L156-L159:

For example, we identify a phased nucleosome array downstream of the TSS. Together with a well-positioned +1 nucleosome and an upstream nucleosome-free region. These findings support a promoter architecture in *Pf* that resembles classical eukaryotic promoters (Bunnik et al. 2014, Kensche et al. 2016).

Results L180-L183:

These new *Pf* nucleosome maps reveal a nucleosome organisation at transcription start sites (TSS) reminiscent of the general eukaryotic chromatin structure, featuring a reported well-positioned +1 nucleosome , an upstream nucleosome-free region (NFR, Bunnik et al. 2014, Kensche et al. 2016), and shown for the first time in *Pf*, a phased nucleosome array downstream of the TSS.

Discussion L412-L421:

Previous analyses of *Pf* chromatin have identified +1 nucleosomes and NFRs (Bunnik et al 2014, Kensche et al. 2016). Here we extend this understanding by demonstrating phased nucleosome array structures throughout the genome. This finding provides evidence for a spatial regulation of nucleosome positioning in Pf, challenging the notion that nucleosome positioning is relatively random in gene bodies (Bunnik et al. 2014, Kensche et al. 2016). Consequently our results contribute to the understanding that Pf exhibits a typical eukaryotic chromatin structure, including well-defined nucleosome positioning at the TSS and regularly spaced nucleosome arrays (Schones et al. 2008; Yuan et al. 2005).

The phrase "poorly understood chromatin architecture" has been modified to "underexplored chromatin architecture" in order to more accurately reflect the potential for further analyses and contributions to the field, while avoiding any potential misinterpretation of an attempt to undervalue previous work.

• Track labels in figures (e.g., Figure 5B) are too small to be legible.

We made the labels bigger.

• Several figures (e.g., Figure 5B, S4B) lack statistical significance tests. Are the differences marked with stars statistically significant or just visually different?

We added statistics to S4B.

Review COMMONS

Differences in 5B were identified by visual inspection. To clarify this, we exchanged the asterisks to arrows in Fig.5B and changed the text in the legend:
Arrows mark descriptive visual differences in nucleosome occupancy.

• Figure S3 includes a small black line on top of the table. Is this an accidental crop?
We checked the figure carefully; however, the black line does not appear in our PDF viewer or on the printed paper

• The authors should state the weaknesses and limitations of this pipeline.
We added a limitation section in discussion, see comments above

Reviewer #1 (Significance (Required)):

• The proposed pipeline is useful and timely. It can benefit research groups willing to analyse MNase-Seq data of complex genomes such as P. falciparum. The tool requires users to have extensive experience in coding as the authors didn't include any clear and explicit codes on how to start processing the data from raw files. Nevertheless, there are multiple tool that can detect nucleosome occupancy and that are not cited by the authors not mention. I have included for the authors a link where a large list of tools for analysis of nucleosome positioning experiments tools/pipelines were developed for (Software to analyse nucleosome positioning experiments - Gene Regulation - Teif Lab). I think it would be useful for the authors to direct the reference this.
We appreciate the reviewer's valuable suggestion. We included a citation to the comprehensive database of nucleosome analysis tools curated by the Teif lab (Shtumpf et al., 2022). We chose to reference only selected tools in addition to this resource rather than listing all individual tools to maintain clarity and avoid overloading the manuscript with numerous citations.
• Despite valid, I still believe that controlling their pipeline by filtering out false positives and including more QC steps at the Inspector stage is strongly needed. That would boost the significance of this pipeline.
We thank the reviewer for the assessment of our study and for recognizing that our MNase-seq analysis pipeline nucDetective can be a useful tool for the chromatin community utilizing MNase-Seq in complex settings.

Reviewer #2 (Evidence, reproducibility and clarity (Required)):

In this manuscript, Holzinger and colleagues have developed a new pipeline to assess chromatin organization in linear space and time. They used this pipeline to reevaluate nucleosome organization in the malaria parasite, P. falciparum. Their analysis revealed typical arrangement of nucleosomes around the transcriptional start site. Furthermore, it further strengthened and refined the connection between specific nucleosome dynamics and epigenetic marks, transcription factor binding sites or transcriptional activity.

Major comments

- I am wondering what is the main selling point of this manuscript is. If it is the development of the nucDetective pipeline, perhaps it would be best to first benchmark it and directly compare it to existing tools on a dataset where nucleosome fussiness, shifting and regularity has been analyzed before. If on the other hand, new insights into Plasmodium chromatin biology is the primary target validation of some of the novel findings would be advantageous (e.g. refinement of TSS positions, relevance of novel motifs, etc).
NucDetective presents a novel pipeline to identify dynamic nucleosome properties within different datasets, like time series or developmental stages, as analysed for the erythrocytic cycle in this manuscript. As such kind of a pipeline, allowing direct comparisons, does not exist for MNase-Seq data, we used the existing analysis and high quality dataset of Kensche et al., to visualize the strong improvements of this kind of analysis. Accordingly, we combined the pipeline development and the reasearch of chromatin structure analysis, being able to showcase the utility of this new pipeline.

- The authors identify a strong positioning of +1 nucleosome by searching for a positioned nucleosomes in the vicinity of the assigned TSS. Given the ill-defined nature of TSSs, this approach sounds logic at first glance. However, given the rather broad search space from -100 till +300bp, I am wondering whether it is a sort of "self-fulfilling prophecy". Conversely, it would be good to validate that this approach indeed helps to refine TSS positions.
We thank the reviewer for raising this important point. We would like to clarify that we do not claim to redefine or precisely determine TSS positions in our study. Instead, we use annotated TSS coordinates as a reference to identify nucleosomes that correspond to the +1 nucleosome, based on their proximity to the TSS.
We selected the search window from -100 to +300 bp to account for known variability in *Pf* TSS annotation. For example, dominant transcription start sites identified by 5'UTR-seq tag clusters can differ by several hundred base pairs within a single time point (Chappell et al., 2020). The broad window thus allows us to capture the principal nucleosome positions near a TSS, even when the TSS itself is imprecise or heterogeneous. Based on the TSS centered plots (Figure 2C and Figure S1B), we reasoned that a window of -100 to +300 is sufficient to capture the majority of the +1 nucleosomes, which would have been missed by using smaller window sizes. This strategy aligns with well-established conventions in yeast chromatin biology, where the +1 nucleosome is defined relative to the TSS (Jiang and Pugh, 2009; Zhang et al. 2011) and commonly used as an anchor point to visualize downstream phased nucleosome arrays and upstream nucleosome-depleted regions (Rossi et al., 2021; Oberbeckmann et al., 2019; Krietenstein et al., 2016 and many more). Accordingly, our approach leverages these accepted standards to interpret nucleosome positioning without re-defining TSS annotations.

- Figure 1C: I am wondering how should the reader interpret the changes in nucleosomal repeat length

changes throughout the cycle. Is linker DNA on average 10 nucleotides shorter at T30 compared to T5 timepoint? If so how could such "dramatic reorganization" be achieved at the molecular level in absence of a known linker DNA-binding protein. More importantly is this observation supported by additional evidence (e.g. dinucleosomal fragment length) or could it be due to slightly different digestion of the chromatin at the different stages or other technical variables?

We thank the reviewer for this insightful question regarding the interpretation of NRL changes across the cell cycle. The reviewer is right in her or his interpretation – linker DNA is on average ~10 bp shorter at T30 than at T5.

To address concerns about additional evidence and potential MNase digestion variability, we now analyzed MNase-seq fragment sizes by shifting mononucleosome peaks of each time point to the canonical 147 bp length, to correct for MNase digestion differences. After this normalisation, dinucleosome fragment length distributions revealed the shortest linker lengths at T30 and T35, whereas T5 and T10 showed longer DNA linkers. These results confirm our previous NRL measurements based on mononucleosomal read distances while controlling for MNase digestion bias.

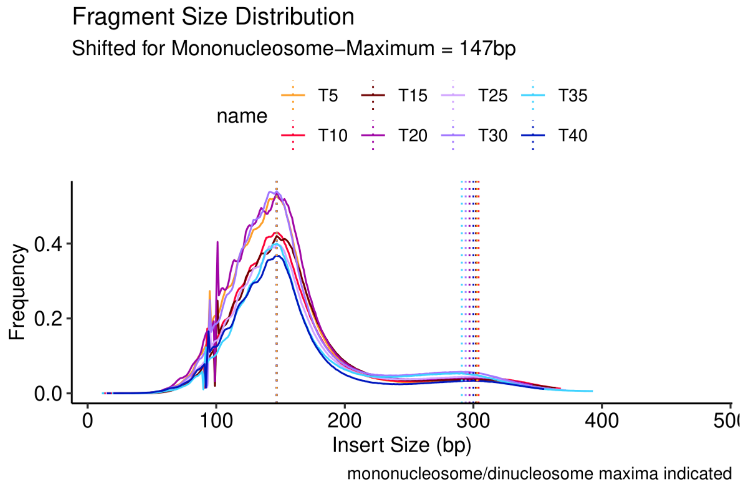

The molecular basis of this reorganization, is still unclear. While linker histone H1 is considered absent in *Plasmodium falciparum*, presence of an uncharacterized linker DNA–binding protein or alternative factors fulfilling a similar role can not be excluded (Gill et al. 2010). However, H1 absence across all developmental stages, fails to explain stage-specific chromatin changes. We hypothesize that Apicomplexans evolved specialized chromatin remodelers to compensate for the missing H1, which may also drive the dynamic NRL changes observed. The low NRL coincides with high transcriptional activity in *Pf* during trophozoite stage is consistent with previous reports linking elevated transcription to reduced NRL in other eukaryotes (Baldi et al. 2018). In addition, the schizont stage involves multiple rounds of DNA replication requiring large histone supplies being produced during that time. It may well be that a high level of histone synthesis and DNA amplification, results in a short time period with increased nucleosome density and shorter NRL, until the system reaches again equilibrium (Beshnova et al. 2014). Although speculative we suggest a model wherein increased transcription promotes elevated nucleosome turnover and re-assembly by specialized remodeling enzymes, combined with high abundance of histones, resulting in higher nucleosome density and decreased NRL. Unfortunately, absolute quantification of nucleosome levels from this MNase-seq dataset is not possible without spike-in controls, which makes it infeasible to test the hypothesis with the available data set (Chen et al. 2016).

Minor comments

- I am wondering whether fuzziness and occupancy changes are truly independent categories. I am asking as both could lead to reduction of the signal at the nucleosome dyad and because they show markedly similar distribution in relation to the TSS and associate with identical epigenetic features (Figure 2B-D). Figure 2A indicates minimal overlap between them, but this could be due to the fact that the criteria to define these subtypes is defined such to place nucleosomes to one or the other category, but at the end they represent two flavors of the same thing.

Indeed, changes in occupancy and fuzziness can appear related because both features may reduce signal intensity at the nucleosome dyad and both are connected to “poor nucleosome positioning”. However, their definitions and measurements are clearly distinct and technically independent. Occupancy reflects the peak height at the nucleosome dyad, while fuzziness quantifies the spread of reads around the peak, measured as the standard deviation of read positions within each nucleosome peak (Jiang and Pugh, 2009; Chen et al., 2013). Although a reduction in occupancy can contribute to increased fuzziness by diminishing the dyad axis signal, fuzziness primarily arises from increased variability in the flanking regions around the nucleosome position center. While this distinction is established in the field, it is also often confused by the concept of well (high occupancy, low fuzziness) and poorly (high fuzziness, low occupancy) positioned nucleosomes, where both of these features are considered.

- Do the authors detect spatial relationship between fuzzy and repositioned/evicted nucleosomes at the level of individual nucleosomes pairs. With other words, can fuzziness be the consequence of repositioning/eviction of the neighboring nucleosome?

In Figure 2A we analyse the spatial overlap of all features to each other. The analysis clearly shows that fuzziness, occupancy changes and position changes occur mostly at distinct spatial sites (overlaps between 3 and 10%, Fig. 2A). Therefore, we suggest that the features correspond to independent processes. Likewise, we do observe an overlap between occupancy and ATAC-seq peaks, but not nucleosome positioning shifts, clearly discriminating different processes.

- Figure 4: enrichment values and measure of statistical significance for the different motifs are missing. Also have there been any other motifs identified.

This information is present in Supplemental Figure S3. Here we show the top 3 hits in each cluster. In the figure legend of Figure 4 we reference to Fig. S3:

L1054 –1055:

“Additional enriched motifs along with the significance of motif enrichment and the fraction of motifs at the respective nucleosome positions are shown in Figure S3”

- The M&M would benefit from some more details, e.g. settings in the piepline, or which fragment sizes were used to map the MNase-seq data?

We included a link to the corresponding Zenodo repository (https://doi.org/10.5281/zenodo.16779899) in the Data and materials availability statement.

The repository contains:

Code (scripts.zip) and annotation of Plasmodium falciparum (Annotation.zip) to reproduce the nucDetective v1.1 (nucDetective-1.1.zip) analysis as done in the research manuscript entitled "Deciphering chromatin architecture and dynamics in Plasmodium falciparum using the nucDetective pipeline".

The folder "output_nucDetective" conains the complete output of the nucDetective analysis pipeline as generated by the "01_nucDetective_profiler.sh" and "02_nucDetective_inspector.sh" scripts.

Review COMMONS

Nucleosome coverage tracks, annotation of nucleosome positions and dynamic nucleosomes are deposited additonally in the folder "Pf_nucleosome_annotation_of_nucDetective".
To make this clearer we added following text to Material and Methods in ”The nucDetective pipeline” section:
Changes in the manuscript (L518-519):
The code, software and annotations used to run the nucDetective pipeline along with the output have been deposited on Zenodo (https://doi.org/10.5281/zenodo.16779899).
which fragment sizes were used to map the MNase-seq data?
The default setting in nucDetective is to use fragment sizes of 140 – 200 bp, which corresponds to the main mono-nucleosome fraction in standard MNase-seq experiments. However, the correct selection of fragment sizes may vary depending on the organism and the variations in MNase-seq protocols. Therefore, the pipeline offers the option of changing the cutoff parameter (--minLen; --maxLen), accordingly. Kensche et al thoroughly tested the best selection of the fragment sizes for the data set, which is used in this manuscript. We agree with their selection and used the same cutoffs (75-175 bp).
This is stated in line 535-536:
The fragments are further filtered to mono-nucleosome sized fragments (here we used 75 – 175 bp)
We changed the text:
The fragments are further filtered to mono-nucleosome sized fragments (default setting 140-200 bp; changed in this study to 75 – 175 bp)
We highlighted other parameters used in this study in the material and methods part.

Reviewer #2 (Significance (Required)):

Overall, the manuscript is well written and findings are clearly and elegantly presented. The manuscript describes a new pipeline to map and analyze MNase-seq data across different stages or conditions, though the broader applicability of the pipeline and advancements over existing tools could be better demonstrated. Importantly, the manuscript make use of this pipeline to provide a refined and likely more accurate view on (the dynamics of) nucleosome positioning over the AT-rich genome of P. falciparum. While these observations make sense they remain rather descriptive/associative and lack further experimental validation. Overall, this manuscript could be interest to both researchers working on chromatin biology and Plasmodium gene-regulation.
We thank the reviewer for the assessment of our study and for recognizing that the results of our MNase-seq analysis pipeline nucDetective contribute to a better understanding of Pf chromatin biology.

Full Revision

Reviewer #3 (Evidence, reproducibility and clarity (Required)):

The manuscript "Deciphering chromatin architecture and dynamics in Plasmodium 2 falciparum using the nucDetective pipeline" describes computational analysis of previously published data of P falciparum chromatin. This work corrects the prevailing view that this parasitic organism has an unusually disorganized chromatin organization, which had been attributed to its high genomic AT content, lack of histone H1, and ancient derivation. The authors show that instead P falciparum has a very typical chromatin organization. Part of the refinement is due to aligning data on +1 nucleosome positions instead of TSSs, which have been poorly mapped. The computational tools corral some useful features, for querying epigenomic structure that make visualization straightforward, especially for fuzzy nucleosomes.

Reviewer #3 (Significance (Required)):

As a computational package this is a nice presentation of fairly central questions. The assessment and display of fuzzy nucleosomes is a nice feature.

We thank the reviewer for the assessment of our study and are pleased that the reviewer acknowledges the value and usability of our pipeline.
