# Supplementary material for: Deciphering chromatin architecture and dynamics in Plasmodium falciparum using the nucDetective pipeline
Source: PLoS Comput Biol. 2026 Jul 27;22(7):e1014557. doi: 10.1371/journal.pcbi.1014557 (PMC13426949; doi:10.1371/journal.pcbi.1014557)
Supplement: Attachment — Submitted filename: 2026-06-25 PF_BioinformaticsPaper ReviewerResponse.docx [file pcbi.1014557.s008.docx]

### Revision of PCOMPBIOL-D-26-00605

### Deciphering chromatin architecture and dynamics in Plasmodium falciparum using the nucDetective pipeline

Dear Vladimir, dear reviewer,

Thank you for allowing us to provide additional analyses as requested. We have addressed all points and believe the manuscript has improved.

The original text from the reviewers is given in black and our answers are provided in blue color. We provide a clean document (2026-06-25_Holzinger manuscript - clean) and a marked document, showing all our changes in blue color (2026-06-25_Holzinger manuscript - marked).

We appreciate the time and effort you devoted to reviewing this manuscript.

Best wishes,

Gernot Längst and team.

…

Please **include the following items** when submitting your revised manuscript:

- **A letter that responds to each point raised by the editor and reviewer(s).** You should upload this letter as a separate file labeled 'Response to Reviewers'. This file does not need to include responses to formatting updates and technical items listed in the 'Journal Requirements' section below. (This document: 2026-06-25 PF_BioinformaticsPaper ReviewerResponse)
- **A marked-up copy of your manuscript that highlights changes made to the original version**. You should upload this as a separate file labeled 'Revised Manuscript with Track Changes'.
  2026-06-25_Holzinger manuscript - clean
- **An unmarked version of your revised paper without tracked changes**. You should upload this as a separate file labeled 'Manuscript'.
  2026-06-25_Holzinger manuscript - marked

…

We look forward to receiving your revised manuscript.

Kind regards,

Vladimir B Teif, Ph.D.
Academic Editor
PLOS Computational Biology

### Editor Comments:

Thank you very much for the opportunity to read in detail your manuscript and responses to previous peer-reviews from Review Commons. I have invited the peer-reviewers who previously reviewed your manuscript at Review Commons to respond**. Reviewer 1 has provided a detailed peer-review, but reviewers 2 and 3 were not available**. Therefore**, I have done my own reading** of the current version of the manuscript and **evaluated your responses to reviewers 2 and 3**. Based on my reading, as well as the response of reviewer 1, which is included separately, this manuscript **requires a minor revision**, as detailed below.

We thank the editor for his thorough assessment of our manuscript and for the previous and current comments by the reviewers. In our revised manuscript, we addressed all points raised by you and Reviewer 1 (see below for a point-by-point response). Additionally, we revised the manuscript to conform to the PLOS Computational Biology style guide.

**1) Please address the minor revision suggestions from Reviewer 1.**

We did. See below.

2) Reviewer 2 asked several relevant questions in relation to the NRL change. I can see your substantial response to this reviewer’s point in the response letter, including **a fragment size histogram plot** that the reviewer asked for. However, this response is **not included in the revised manuscript**. I think the NRL change is an important biological insight of this work. Indeed, we have already cited this preprint in our recent review article and discussed this NRL effect there (Bikova et al (2026) Nucleic Acids Res 54, gkag074, <https://academic.oup.com/nar/article/54/5/gkag074/8506906>). Since the NRL effect is quite central to this work, I think **the discussion in response to Reviewer 2 needs to be moved to the main manuscript, and it can be further expanded.**

We thank the editor for his assessment. Indeed, the change of NRL during development and in different cell types is an interesting phenomenon, as you discuss in depth in your review article, which was not available when we originally wrote our manuscript. Since it summarises the current knowledge in the field very well, we decided to cite it in our Discussion regarding NRL changes during development in other organisms and their association with transcription. We have now expanded our discussion in the manuscript based on our response to Reviewer 2. It now reads:

L576-604: […] The observed variation corresponds to an approximately 9 bp change in average linker DNA length, indicating substantial reorganization of internucleosomal spacing during parasite development comparable to developmental NRL changes reported in other differentiated eukaryotic cells (Bikova et al. 2026) despite the apparent absence of a canonical linker histone H1.

Notably, NRL changes followed a continuous pattern throughout the developmental cycle, decreasing from the ring stage to the trophozoite stage before increasing again during schizogony. The shortest NRL was observed in the trophozoite stage of *Pf,* which coincides with elevated transcriptional activity and significant opening of chromatin (Bunnik et al. 2014; Ay et al. 2014; Ponts et al. 2010; Lu et al. 2017; Kensche et al. 2016). Conversely, the longest NRL is observed during the ring stage, shortly after erythrocyte invasion, when transcriptional activity is very minimal (Kensche et al. 2016; Lu et al. 2017). This inverse relationship between NRL and transcriptional activity aligns with findings across a wide range of eukaryotic systems, in which highly transcribed cell types and genomic regions generally exhibit shorter nucleosome spacing (Baldi et al. 2018; Bikova et al. 2026). The developmental NRL dynamics observed in *Pf,* therefore, suggest that modulation of nucleosome spacing may represent a conserved organizational principle of active chromatin despite the highly divergent chromatin landscape of apicomplexan parasites.

The molecular basis of stage-specific NRL reorganization remains unclear. While linker histone H1 is considered absent in *Pf*, the presence of an as-yet uncharacterized linker DNA-binding protein or alternative factors fulfilling a similar role cannot be excluded (Gill et al. 2010). However, the absence of H1 across all developmental stages cannot explain stage-specific changes in NRL. We hypothesize that Apicomplexans have evolved specialized chromatin remodelers to compensate for the missing H1, which may also drive the dynamic NRL changes observed. Furthermore, the schizont stage involves multiple rounds of DNA replication requiring large histone supplies. It is possible that a high level of histone synthesis and DNA amplification transiently increases nucleosome density and shortens NRL until the system reaches equilibrium (Beshnova et al. 2014). We therefore suggest a model in which increased transcription promotes higher nucleosome turnover and reassembly by specialized remodeling enzymes, combined with high histone abundance, resulting in greater nucleosome density and decreased NRL.

3) As part of the NRL discussion, Reviewer 2 asked to check whether **any differences between developmental stages are observed for the DNA fragment size distribution, including dinucleosome sizes**. This is an interesting question, and it seems that it has **not been addressed in the revised manuscript**. To see better the differences in fragment size distributions of different samples, I suggest to **modify the Y-axis scale of the figure which you have provided in response to Reviewer 2** (e.g. you can make it log-normal scale, etc) and **include this figure in the revised manuscript or supplementary materials**. It will be also good to **show the original phasogram curves for each developmental stage in a similar overlay format in a separate figure**, to be included either in the main manuscript or in the supplementary materials. This will address the concerns of Reviewer 2 regarding biological significance of the NRL results versus MNase-seq digestion variability.

We included an inset plot of the fragment size distribution with a zoom on the dinucleosome area, as we believe this presentation best conveys the overall picture. We also now include an overlay of the smoothed phasograms for each developmental stage. Since we view both figures more as controls for the main NRL figure, we moved them into the supplementary materials as new subfigures S1C and S1D. We also added corresponding sections in the Results and Discussion.

Results:

L219: To quantify the average nucleosome repeat length (NRL) at each timepoint, we used a phasogram-based approach (S1C Fig). The largest NRL occurs in the ring stage at T5 (185 bp) and gradually decreases towards the trophozoite stage at T30 (176 bp), resulting in a total change of approximately 10 bp throughout the developmental cycle. To account for potential variability in MNase digestion across timepoints, we normalised fragment size distributions by shifting mononucleosome peaks to the canonical 147 bp, then assessed dinucleosome fragment length distributions after this adjustment. This showed the shortest linker lengths at T30 and T35, while T5 and T10 exhibited longer DNA linkers (S1D Fig), aligning with our phasogram-based NRL measurements (S1C Fig).

For Discussion see our answer to point 3).

4) In the **Methods section**, please **add technical details of where the Kensche et al data was obtained from** (GEO accession number), and **how was it processed** (e.g. whether separate SRA runs corresponding to the same developmental stage were merged, what was the alignment rate, etc).

We added a sentence detailing technical aspects of the Kensche dataset. We also included a new supplementary table S2 with fragment count statistics throughout the pipeline, which are automatically generated by the pipeline. We mentioned this in the Methods section as well.

The Material and Methods section now reads:

L626: MNase-seq data from Kensche et al. [31] (GEO accession number GSE66185) were obtained from the Sequence Read Archive (SRA). Separate SRA runs corresponding to the same timepoint in IDC were merged (see SRX codes in S2 Table) and converted to fastq files using the SRA toolkit (https://github.com/ncbi/sra-tools).

L655: The fragment statistics are assessed at every stage (S2 Table).

5) The **Swissknife package needs a reference** with names of authors, as listed at their web site: <https://fmicompbio.github.io/swissknife/reference/index.html>. It can be also noted that this package is using an algorithm that is slightly different from the algorithm in Valouev et al, so it is not a simple implementation of Valouev et al.

We included the reference and noted the algorithmic deviations. It now reads:

L659: Nucleosome repeat lengths (NRLs) are calculated based on a phasogram approach originally described in (Valouev et al. 2011) and implemented with a slightly different algorithm in the swissknife (v0.40) package (Stadler et al. 2023).

6**) Line 620** mentions “Custom R script”. Please **make this script publicly available**.

We now include the liftover script on GitHub for the manuscript analysis ([www.github.com/SimHolz/Holzinger_et_al_2025](http://www.github.com/SimHolz/Holzinger_et_al_2025)).

L743: …by a custom R script (available at www.github.com/SimHolz/Holzinger_et_al_2025).

### Journal Requirements:

1) We note that there **were multiple versions** of 2025-11-27_Holzinger manuscript +revision.pdf in your submission's file inventory. We have **removed the older file(s),** retaining the most recent version(s) for editorial review. Please **double check your submission file inventory** and let us know if **any files appear to be outdated or absent**.

We will do upon upload.

2) We ask that a manuscript source file is provided at Revision. Please upload your manuscript file as a .doc, .docx, .rtf or .tex. If you are providing a .tex file, please upload it under the item type u2018LaTeX Source Fileu2019 and leave your .pdf version as the item type u2018Manuscriptu2019.

ok. ?

3) **Please provide an Author Summary.** This should appear in your manuscript between the Abstract (if applicable) and the Introduction, and should be 150-200 words long. The aim should be to make your findings accessible to a wide audience that includes both scientists and non-scientists. Sample summaries can be found on our website under Submission Guidelines: <https://journals.plos.org/ploscompbiol/s/submission-guidelines#loc-parts-of-a-submission>

The author summary is provided and reads:

Understanding how DNA is packaged inside cells is crucial for studying gene regulation during development. In eukaryotes, DNA wraps around protein spools to form nucleosomes. The precise positioning of these nucleosomes along the genome serves as a key layer of gene regulation. We developed nucDetective, a computational pipeline that maps nucleosomes across the genome and compares their organisation between samples. It measures nucleosome positioning, spacing, occupancy, fuzziness, and array regularity. We applied nucDetective to the malaria parasite *Plasmodium falciparum* and analysed nucleosome organisation throughout its developmental stages inside human red blood cells. Contrary to the current view that this parasite has irregular chromatin, we discovered regular phased nucleosome arrays downstream of transcription start sites. Along with the established +1 nucleosome and a nucleosome-depleted region upstream, these nucleosome arrays form a promoter structure similar to that of other eukaryotes. Nucleosome spacing varied between developmental stages, and nucleosomal rearrangements occurred in intergenic regulatory regions. These dynamic nucleosomes were associated with histone modifications, histone variants, DNA accessibility, gene expression, and transcription factor binding sites. Our findings reveal an underappreciated level of specific and dynamic chromatin organisation in the malaria parasite that aids understanding of developmental processes and helps identify therapeutic targets.

Author Summary length: 198 words

4) Please upload **all main figures [Figures 1-5] as separate Figure files in .tif or .eps format.** For more information about how to convert and format your figure files please see our guidelines: 
<https://journals.plos.org/ploscompbiol/s/figures>

All main Figures will be uploaded as .tif files.

5) Please **upload a copy of [Figures 1-5] which you refer to in your text on pages** [6, 8, 9, 10, and 11]. Or, **if the figure is no longer to be included** as part of the submission please **remove all reference to it** within the text.

Upon submission, we will clean up and upload only the current, updated figures.

6) We have noticed that you have uploaded Supporting Information files, but you have not included a list of legends. Please **add a full list of legends for your Supporting Information files** after the references list.

The legends are now included.

7) We notice that your **supplementary Figures [1-4] are included in the manuscript file**. Please **remove** them and **upload them with the file type 'Supporting Information'**. Please **ensure that each Supporting Information file has a legend listed in the manuscript** after the references list.

The supplementary Figures and Tables will be uploaded separately.

8) Please **amend your detailed Financial Disclosure statement**. This is published with the article. It must therefore be **completed in full sentences and contain the exact wording you wish to be published**.

1) **State the initials, alongside each funding source, of each author to receive each grant**. For example: "This work was supported by the National Institutes of Health (####### to AM; ###### to CJ) and the National Science Foundation (###### to AM)."

Done

2) **State what role the funders took in the study. If the funders had no role in your study, please state**: "The funders had no role in study design, data collection and analysis, decision to publish, or preparation of the manuscript."

Done.

3) **If any authors received a salary from any of your funders, please state which authors and which funders**.

Done.

### Reviewer Comments:

### Reviewer #1:

I thank the authors for their detailed responses and for the substantial revisions made to the manuscript. The revised version is **considerably improved**, and **many of my original concerns have been addressed**. In particular, I appreciate the clarification regarding previous reports of +1 nucleosome positioning in Plasmodium falciparum, the improved discussion of methodological limitations, the additional analyses provided throughout the manuscript, and the efforts made to improve reproducibility through the deposition of code, annotations, and outputs in a permanent repository.

We thank the reviewer for their time and careful evaluation of our revised manuscript and are pleased that they found it significantly improved. We also sincerely value the reviewer’s constructive comments, critical questions, and valid concerns, which have helped us to strengthen the manuscript considerably.

1. Clarification of +1 nucleosome positioning in P. falciparum

**Assessment: Addressed**

The manuscript now appropriately acknowledges previous studies reporting +1 nucleosomes and nucleosome-free regions in P. falciparum. The revised text more accurately defines the novelty of the present study as the identification of phased downstream nucleosome arrays rather than the discovery of the +1 nucleosome itself.

I also appreciate the additional analyses examining the relationship between +1 nucleosome positioning and gene expression, which are consistent with previous observations in the field.

2. Reference nucleosome numbers

**Assessment: Addressed**

The authors now provide additional context regarding the total number of nucleosomes identified and compare these values with previous studies. This improves the interpretation of the dataset and clarifies how the reference nucleosome set was defined.

3. Use of mono-nucleosome fragments

**Assessment: Addressed**

The authors clearly explain that the improved nucleosome maps result not only from fragment selection but also from differences in preprocessing and alignment strategies. They also acknowledge that the pipeline has been optimized for mono-nucleosome analysis and that performance on di- and tri-nucleosome fragments remains untested.

I consider this concern adequately addressed.

4. Genome-wide occupancy and chromosomal distribution

**Assessment: Largely addressed**

The revised manuscript now discusses the accumulation of dynamic nucleosome features at centromeric regions and the depletion observed at chromosome ends. The additional supplementary analyses improve interpretation of these observations and address most of my concerns regarding genome-wide distribution.

5. Dependence on DANPOS

Assessment: Addressed

The relationship between nucDetective and DANPOS is now much clearer. The authors appropriately distinguish between existing tools incorporated into the workflow and the original components developed as part of nucDetective.

6. Reproducibility and data availability

**Assessment: Addressed**

The addition of the Zenodo repository, together with the clarification regarding scripts, annotations, pipeline outputs, and reproducibility resources, substantially improves transparency and reproducibility.

**Remaining concerns**

7. Quality control and validation of dynamic nucleosome detection

Assessment: Partially addressed

I appreciate the expanded description of quality-control procedures and the rationale provided by the authors regarding insert-size distributions, TSS profiles, PCA structure, replicate consistency, and visual inspection of nucleosome profiles.

However, **my original concern regarding quality control within the Inspector workflow itself remains only partially addressed.** The response primarily explains why the authors consider the current workflow sufficient, but **additional QC metrics or validation procedures for downstream dynamic nucleosome calls have not been implemented**.

I think it is important to distinguish between quality control of MNase-seq data generation and processing, and **validation of the biological conclusions derived from dynamic nucleosome detection**. While the manuscript now better documents the former, the latter **remains less developed**.

Similarly, the current strategy of **selecting the 20% best-positioned nucleosomes** based on fuzziness represents a conservative filtering approach, but it **does not directly estimate false-positive rates or independently validate dynamic nucleosome calls**. I understand the authors’ argument that no accepted gold-standard dataset exists for this purpose. Nevertheless, I believe the manuscript should more clearly emphasize that **the identified loci represent high-confidence candidate dynamic nucleosomes rather than a formally validated set of differential nucleosome events**.

To address the remaining concerns, we included a sentence in the Results that explains the rationale behind the use of the variance-based prioritization strategy.

L237: As MNase-seq data sets are often limited by sequencing depth and replicate number required for formal differential analysis, we implemented a variance-based prioritization strategy to screen for dynamic nucleosomes, analogues to similar strategies that are used to define super enhancers or unstable nucleosomes (Whyte et al. 2013; Wernig-Zorc et al. 2024).

Furthermore, we emphasise later in the Results that “dynamic nucleosomes” is used throughout the manuscript as an operational term for the conservative, variance-prioritized set of high-confidence nucleosome changes identified by Inspector. This avoids repeated use of “candidate” throughout the manuscript while making the scope and limitations of the calls explicit at their first introduction.

L255: For clarity, we use the term “dynamic nucleosomes” throughout the manuscript to refer to this conservative, variance-prioritized set of high-confidence nucleosome changes, rather than a formally FDR-controlled set of differential nucleosome events. To assess the biological relevance and information content of these extracted features, a Principal Component Analysis (PCA) was employed on each set of candidate dynamic nucleosomes.

We also added to the Limitations Section:

L538: Accordingly, dynamic nucleosomes identified by nucDetective are considered high-confidence candidate loci, ranked by variance. This method offers a systematic screening strategy for detecting significant chromatin changes and generating biologically relevant hypotheses, but it does not directly assess false-positive rates or substitute for independent statistical validation.

8. Statistical support for time-series analyses

Assessment: Partially addressed

The authors now clarify that nucDetective is intended primarily as a screening framework and that dynamic nucleosomes are identified through variance-based ranking rather than formal statistical testing. The addition of a dedicated limitations section is welcome and improves transparency.

However, this clarification also highlights an important limitation of the current approach. **Dynamic nucleosomes are not identified using a statistical framework that explicitly models biological variance across conditions.** Consequently, **the strength of evidence supporting differential nucleosome behaviour remains difficult to assess quantitatively**.

I do not necessarily view this as a fatal limitation, particularly given the challenges associated with MNase-seq experiments and the limited number of biological replicates available in many published datasets. However, **I recommend that the authors continue to moderate their wording throughout the manuscript** and **ensure that dynamic nucleosomes are presented within the context of a variance-based prioritization strategy rather than a statistically validated differential analysis**.

Please refer to our response to point 7, which also covers this point.

9. Sequence bias and gDNA normalization

Assessment: Partially addressed

The authors now clearly explain the use of gDNA normalization and provide a rationale for not incorporating sequence normalization into the general workflow. The added discussion improves transparency.

Nevertheless, given the extreme AT-rich composition of the P. falciparum genome and the well-established sequence preferences of MNase, I still consider this an important limitation. The issue is now acknowledged in the Discussion, which is an improvement, but **readers should be reminded that the reported nucleosome profiles are not corrected for sequence-specific biases**.

To clarify, we included in the Result section and the figure legend of Figure 1:

L166: Nucleosome profiles were not corrected for sequence-specific MNase biases, as the downstream analysis is focused on direct comparison between timepoints (see Discussion for details).

L177: […]. The reported nucleosome profiles were not corrected by gDNA or MNase-sequence-bias normalization. It provides centered nucleosome coverage tracks (T5-T40 colored coverage tracks) […]

We also address this point by now explicitly mentioning when correcting for MNase sequence preferences.

L421: Additionally, as it is not included in the nucDetective pipeline, we normalized the nucleosome occupancy by MNase treated gDNA to account for potential MNase sequence preferences.

10. Statistical support for specific figures

**Assessment: Addressed**

The corrections regarding figure references, the addition of statistical support for nucleosome repeat length analyses, and the clarification that Figure 5B highlights visual observations rather than statistical significance address my concerns regarding figure interpretation.

Overall assessment

The manuscript has improved substantially and addresses the majority of the concerns raised in my original review. The remaining issues primarily concern validation and interpretation rather than the biological observations themselves.

In particular, I believe **the manuscript would benefit from a clearer distinction between**: **(i) quality control of MNase-seq data processing**, **(ii) identification of candidate dynamic nucleosomes**, and **(iii) statistical validation of differential nucleosome behaviour**. At present, these concepts occasionally overlap in the discussion of pipeline performance.

Overall, nucDetective appears to be a useful and reproducible framework for identifying and prioritizing candidate dynamic nucleosomes across complex experimental designs. However, I encourage the authors to further **moderate statements regarding dynamic nucleosome detection** and to **emphasize the exploratory nature of the approach where appropriate.**

With these revisions, I believe the manuscript would provide a valuable contribution both as a computational resource and as an updated view of chromatin organization in Plasmodium falciparum.

We thank the Editor and the Reviewer for their input.

With best regards

Simon Holzinger, Uwe Schwartz and Gernot Längst
