# Supplementary material for: Deciphering chromatin architecture and dynamics in Plasmodium falciparum using the nucDetective pipeline
Source: PLoS Comput Biol. 2026 Jul 27;22(7):e1014557. doi: 10.1371/journal.pcbi.1014557 (PMC13426949; doi:10.1371/journal.pcbi.1014557)
Supplement: Peer review history

# Author comment: Author response to Decision Letter 0

**DOI:** 10.1371/journal.pcbi.1014557.r001

22 Mar 2026

Attachment Submitted filename: 11-25 full-revision RC-2025-03175.pdf

# Peer review: Decision Letter 0

**DOI:** 10.1371/journal.pcbi.1014557.r002

**Authors:** Vladimir Teif, Shaun Mahony

9 Jun 2026

PCOMPBIOL-D-26-00605

Deciphering chromatin architecture and dynamics in Plasmodium falciparum using the nucDetective pipeline

PLOS Computational Biology

Dear Dr. Laengst,

Thank you for submitting your manuscript to PLOS Computational Biology. After careful consideration, we feel that it has merit but does not fully meet PLOS Computational Biology's publication criteria as it currently stands. Therefore, we invite you to submit a revised version of the manuscript that addresses the points raised during the review process.

Please submit your revised manuscript by Aug 09 2026 11:59PM. If you will need more time than this to complete your revisions, please reply to this message or contact the journal office at ploscompbiol@plos.org. When you're ready to submit your revision, log on to https://www.editorialmanager.com/pcompbiol/ and select the 'Submissions Needing Revision' folder to locate your manuscript file.

Please include the following items when submitting your revised manuscript:

* A letter that responds to each point raised by the editor and reviewer(s). You should upload this letter as a separate file labeled 'Response to Reviewers'. This file does not need to include responses to formatting updates and technical items listed in the 'Journal Requirements' section below.

* A marked-up copy of your manuscript that highlights changes made to the original version. You should upload this as a separate file labeled 'Revised Manuscript with Track Changes'.

* An unmarked version of your revised paper without tracked changes. You should upload this as a separate file labeled 'Manuscript'.

If you would like to make changes to your financial disclosure, competing interests statement, or data availability statement, please make these updates within the submission form at the time of resubmission. Guidelines for resubmitting your figure files are available below the reviewer comments at the end of this letter.

As the corresponding author, your ORCID iD is verified in the submission system and will appear in the published article. PLOS supports the use of ORCID, and we encourage all coauthors to register for an ORCID iD and use it as well. Please encourage your coauthors to verify their ORCID iD within the submission system before final acceptance, as unverified ORCID iDs will not appear in the published article. Only the individual author can complete the verification step; PLOS staff cannot verify ORCID iDs on behalf of authors.

We look forward to receiving your revised manuscript.

Kind regards,

Vladimir B Teif, Ph.D.

Academic Editor

PLOS Computational Biology

Shaun Mahony

Section Editor

PLOS Computational Biology

**Additional Editor Comments (if provided):**

Thank you very much for the opportunity to read in detail your manuscript and responses to previous peer-reviews from Review Commons. I have invited the peer-reviewers who previously reviewed your manuscript at Review Commons to respond. Reviewer 1 has provided a detailed peer-review, but reviewers 2 and 3 were not available. Therefore, I have done my own reading of the current version of the manuscript and evaluated your responses to reviewers 2 and 3. Based on my reading, as well as the response of reviewer 1, which is included separately, this manuscript requires a minor revision, as detailed below.

1) Please address the minor revision suggestions from Reviewer 1.

2) Reviewer 2 asked several relevant questions in relation to the NRL change. I can see your substantial response to this reviewer’s point in the response letter, including a fragment size histogram plot that the reviewer asked for. However, this response is not included in the revised manuscript. I think the NRL change is an important biological insight of this work. Indeed, we have already cited this preprint in our recent review article and discussed this NRL effect there (Bikova et al (2026) Nucleic Acids Res 54, gkag074, https://academic.oup.com/nar/article/54/5/gkag074/8506906). Since the NRL effect is quite central to this work, I think the discussion in response to Reviewer 2 needs to be moved to the main manuscript, and it can be further expanded.

3) As part of the NRL discussion, Reviewer 2 asked to check whether any differences between developmental stages are observed for the DNA fragment size distribution, including dinucleosome sizes. This is an interesting question, and it seems that it has not been addressed in the revised manuscript. To see better the differences in fragment size distributions of different samples, I suggest to modify the Y-axis scale of the figure which you have provided in response to Reviewer 2 (e.g. you can make it log-normal scale, etc) and include this figure in the revised manuscript or supplementary materials. It will be also good to show the original phasogram curves for each developmental stage in a similar overlay format in a separate figure, to be included either in the main manuscript or in the supplementary materials. This will address the concerns of Reviewer 2 regarding biological significance of the NRL results versus MNase-seq digestion variability.

4) In the Methods section, please add technical details of where the Kensche et al data was obtained from (GEO accession number), and how was it processed (e.g. whether separate SRA runs corresponding to the same developmental stage were merged, what was the alignment rate, etc).

5) The Swissknife package needs a reference with names of authors, as listed at their web site: https://fmicompbio.github.io/swissknife/reference/index.html. It can be also noted that this package is using an algorithm that is slightly different from the algorithm in Valouev et al, so it is not a simple implementation of Valouev et al.

6) Line 620 mentions “Custom R script”. Please make this script publicly available.

Best regards,

Vladimir Teif

**Journal Requirements:**

1) We note that there were multiple versions of 2025-11-27_Holzinger manuscript +revision.pdf in your submission's file inventory. We have removed the older file(s), retaining the most recent version(s) for editorial review. Please double check your submission file inventory and let us know if any files appear to be outdated or absent.

2) We ask that a manuscript source file is provided at Revision. Please upload your manuscript file as a .doc, .docx, .rtf or .tex. If you are providing a .tex file, please upload it under the item type u2018LaTeX Source Fileu2019 and leave your .pdf version as the item type u2018Manuscriptu2019.

3) Please ensure that you provide a single, cohesive .tex source file for your LaTeX revision. You may upload this file as the item type 'LaTeX Source File.' Please also ensure that you are making any formatting changes to both your .tex file and the PDF of your manuscript. If you have any questions, please contact customercare@plos.org. You can find our LaTeX guidelines here: https://journals.plos.org/ploscompbiol/s/latex

3) Please provide an Author Summary. This should appear in your manuscript between the Abstract (if applicable) and the Introduction, and should be 150-200 words long. The aim should be to make your findings accessible to a wide audience that includes both scientists and non-scientists. Sample summaries can be found on our website under Submission Guidelines:

https://journals.plos.org/ploscompbiol/s/submission-guidelines#loc-parts-of-a-submission

4) Please upload all main figures [Figures 1-5] as separate Figure files in .tif or .eps format. For more information about how to convert and format your figure files please see our guidelines:

https://journals.plos.org/ploscompbiol/s/figures

5) Please upload a copy of [Figures 1-5] which you refer to in your text on pages [6, 8, 9, 10, and 11]. Or, if the figure is no longer to be included as part of the submission please remove all reference to it within the text.

6) We have noticed that you have uploaded Supporting Information files, but you have not included a list of legends. Please add a full list of legends for your Supporting Information files after the references list.

7) We notice that your supplementary Figures [1-4] are included in the manuscript file. Please remove them and upload them with the file type 'Supporting Information'. Please ensure that each Supporting Information file has a legend listed in the manuscript after the references list.

8) Please amend your detailed Financial Disclosure statement. This is published with the article. It must therefore be completed in full sentences and contain the exact wording you wish to be published.

1) State the initials, alongside each funding source, of each author to receive each grant. For example: "This work was supported by the National Institutes of Health (####### to AM; ###### to CJ) and the National Science Foundation (###### to AM)."

2) State what role the funders took in the study. If the funders had no role in your study, please state: "The funders had no role in study design, data collection and analysis, decision to publish, or preparation of the manuscript."

3) If any authors received a salary from any of your funders, please state which authors and which funders.

**Reviewers' comments:**

Reviewer's Responses to Questions

**Comments to the Authors:**

**Please note here if the review is uploaded as an attachment.**

Reviewer #1: I thank the authors for their detailed responses and for the substantial revisions made to the manuscript. The revised version is considerably improved, and many of my original concerns have been addressed. In particular, I appreciate the clarification regarding previous reports of +1 nucleosome positioning in Plasmodium falciparum, the improved discussion of methodological limitations, the additional analyses provided throughout the manuscript, and the efforts made to improve reproducibility through the deposition of code, annotations, and outputs in a permanent repository.

Below I provide a point-by-point assessment of the revised manuscript.

1. Clarification of +1 nucleosome positioning in P. falciparum

Assessment: Addressed

The manuscript now appropriately acknowledges previous studies reporting +1 nucleosomes and nucleosome-free regions in P. falciparum. The revised text more accurately defines the novelty of the present study as the identification of phased downstream nucleosome arrays rather than the discovery of the +1 nucleosome itself.

I also appreciate the additional analyses examining the relationship between +1 nucleosome positioning and gene expression, which are consistent with previous observations in the field.

2. Reference nucleosome numbers

Assessment: Addressed

The authors now provide additional context regarding the total number of nucleosomes identified and compare these values with previous studies. This improves the interpretation of the dataset and clarifies how the reference nucleosome set was defined.

3. Use of mono-nucleosome fragments

Assessment: Addressed

The authors clearly explain that the improved nucleosome maps result not only from fragment selection but also from differences in preprocessing and alignment strategies. They also acknowledge that the pipeline has been optimized for mono-nucleosome analysis and that performance on di- and tri-nucleosome fragments remains untested.

I consider this concern adequately addressed.

4. Genome-wide occupancy and chromosomal distribution

Assessment: Largely addressed

The revised manuscript now discusses the accumulation of dynamic nucleosome features at centromeric regions and the depletion observed at chromosome ends. The additional supplementary analyses improve interpretation of these observations and address most of my concerns regarding genome-wide distribution.

5. Dependence on DANPOS

Assessment: Addressed

The relationship between nucDetective and DANPOS is now much clearer. The authors appropriately distinguish between existing tools incorporated into the workflow and the original components developed as part of nucDetective.

6. Reproducibility and data availability

Assessment: Addressed

The addition of the Zenodo repository, together with the clarification regarding scripts, annotations, pipeline outputs, and reproducibility resources, substantially improves transparency and reproducibility.

Remaining concerns

7. Quality control and validation of dynamic nucleosome detection

Assessment: Partially addressed

I appreciate the expanded description of quality-control procedures and the rationale provided by the authors regarding insert-size distributions, TSS profiles, PCA structure, replicate consistency, and visual inspection of nucleosome profiles.

However, my original concern regarding quality control within the Inspector workflow itself remains only partially addressed. The response primarily explains why the authors consider the current workflow sufficient, but additional QC metrics or validation procedures for downstream dynamic nucleosome calls have not been implemented.

I think it is important to distinguish between quality control of MNase-seq data generation and processing, and validation of the biological conclusions derived from dynamic nucleosome detection. While the manuscript now better documents the former, the latter remains less developed.

Similarly, the current strategy of selecting the 20% best-positioned nucleosomes based on fuzziness represents a conservative filtering approach, but it does not directly estimate false-positive rates or independently validate dynamic nucleosome calls. I understand the authors’ argument that no accepted gold-standard dataset exists for this purpose. Nevertheless, I believe the manuscript should more clearly emphasize that the identified loci represent high-confidence candidate dynamic nucleosomes rather than a formally validated set of differential nucleosome events.

8. Statistical support for time-series analyses

Assessment: Partially addressed

The authors now clarify that nucDetective is intended primarily as a screening framework and that dynamic nucleosomes are identified through variance-based ranking rather than formal statistical testing. The addition of a dedicated limitations section is welcome and improves transparency.

However, this clarification also highlights an important limitation of the current approach. Dynamic nucleosomes are not identified using a statistical framework that explicitly models biological variance across conditions. Consequently, the strength of evidence supporting differential nucleosome behaviour remains difficult to assess quantitatively.

I do not necessarily view this as a fatal limitation, particularly given the challenges associated with MNase-seq experiments and the limited number of biological replicates available in many published datasets. However, I recommend that the authors continue to moderate their wording throughout the manuscript and ensure that dynamic nucleosomes are presented within the context of a variance-based prioritization strategy rather than a statistically validated differential analysis.

9. Sequence bias and gDNA normalization

Assessment: Partially addressed

The authors now clearly explain the use of gDNA normalization and provide a rationale for not incorporating sequence normalization into the general workflow. The added discussion improves transparency.

Nevertheless, given the extreme AT-rich composition of the P. falciparum genome and the well-established sequence preferences of MNase, I still consider this an important limitation. The issue is now acknowledged in the Discussion, which is an improvement, but readers should be reminded that the reported nucleosome profiles are not corrected for sequence-specific biases.

10. Statistical support for specific figures

Assessment: Addressed

The corrections regarding figure references, the addition of statistical support for nucleosome repeat length analyses, and the clarification that Figure 5B highlights visual observations rather than statistical significance address my concerns regarding figure interpretation.

Overall assessment

The manuscript has improved substantially and addresses the majority of the concerns raised in my original review. The remaining issues primarily concern validation and interpretation rather than the biological observations themselves.

In particular, I believe the manuscript would benefit from a clearer distinction between: (i) quality control of MNase-seq data processing, (ii) identification of candidate dynamic nucleosomes, and (iii) statistical validation of differential nucleosome behaviour. At present, these concepts occasionally overlap in the discussion of pipeline performance.

Overall, nucDetective appears to be a useful and reproducible framework for identifying and prioritizing candidate dynamic nucleosomes across complex experimental designs. However, I encourage the authors to further moderate statements regarding dynamic nucleosome detection and to emphasize the exploratory nature of the approach where appropriate.

With these revisions, I believe the manuscript would provide a valuable contribution both as a computational resource and as an updated view of chromatin organization in Plasmodium falciparum.

**********

**Have the authors made all data and (if applicable) computational code underlying the findings in their manuscript fully available?**

The PLOS Data policy requires authors to make all data and code underlying the findings described in their manuscript fully available without restriction, with rare exception (please refer to the Data Availability Statement in the manuscript PDF file). The data and code should be provided as part of the manuscript or its supporting information, or deposited to a public repository. For example, in addition to summary statistics, the data points behind means, medians and variance measures should be available. If there are restrictions on publicly sharing data or code —e.g. participant privacy or use of data from a third party—those must be specified.

Reviewer #1: Yes

**********

PLOS authors have the option to publish the peer review history of their article (what does this mean?). If published, this will include your full peer review and any attached files.

If you choose “no”, your identity will remain anonymous but your review may still be made public.

**Do you want your identity to be public for this peer review?**  For information about this choice, including consent withdrawal, please see our Privacy Policy.

Reviewer #1: **Yes:**  Ibtissam Jabre

[NOTE: If reviewer comments were submitted as an attachment file, they will be attached to this email and accessible via the submission site. Please log into your account, locate the manuscript record, and check for the action link "View Attachments". If this link does not appear, there are no attachment files.]

**Figure resubmission:**

-->While revising your submission, we strongly recommend that you use PLOS’s NAAS tool (https://ngplosjournals.pagemajik.ai/artanalysis) to test your figure files. NAAS can convert your figure files to the TIFF file type and meet basic requirements (such as print size, resolution), or provide you with a report on issues that do not meet our requirements and that NAAS cannot fix.-->

After uploading your figures to PLOS’s NAAS tool - https://ngplosjournals.pagemajik.ai/artanalysis, NAAS will process the files provided and display the results in the "Uploaded Files" section of the page as the processing is complete. If the uploaded figures meet our requirements (or NAAS is able to fix the files to meet our requirements), the figure will be marked as "fixed" above. If NAAS is unable to fix the files, a red "failed" label will appear above. When NAAS has confirmed that the figure files meet our requirements, please download the file via the download option, and include these NAAS processed figure files when submitting your revised manuscript.

**Reproducibility:**

To enhance the reproducibility of your results, we recommend that authors of applicable studies deposit laboratory protocols in protocols.io, where a protocol can be assigned its own identifier (DOI) such that it can be cited independently in the future. Additionally, PLOS ONE offers an option to publish peer-reviewed clinical study protocols. Read more information on sharing protocols at https://plos.org/protocols?utm_medium=editorial-email&utm_source=authorletters&utm_campaign=protocols

# Author comment: Author response to Decision Letter 1

**DOI:** 10.1371/journal.pcbi.1014557.r003

30 Jun 2026

Attachment Submitted filename: 2026-06-25 PF_BioinformaticsPaper ReviewerResponse.docx

# Editor report: Decision Letter 1

**DOI:** 10.1371/journal.pcbi.1014557.r004

**Authors:** Vladimir Teif, Shaun Mahony

9 Jul 2026

Dear Dr. Längst,

Thank you very much for the revised version of your manuscript. We are pleased to inform you that your manuscript 'Deciphering chromatin architecture and dynamics in Plasmodium falciparum using the nucDetective pipeline' has been provisionally accepted for publication in PLOS Computational Biology.

Before your manuscript can be formally accepted you will need to complete some formatting changes, which you will receive in a follow up email. A member of our team will be in touch with a set of requests.

Please note that your manuscript will not be scheduled for publication until you have made the required changes, so a swift response is appreciated.

IMPORTANT: The editorial review process is now complete. PLOS will only permit corrections to spelling, formatting or significant scientific errors from this point onwards. Requests for major changes, or any which affect the scientific understanding of your work, will cause delays to the publication date of your manuscript.

Should you, your institution's press office or the journal office choose to press release your paper, you will automatically be opted out of early publication. We ask that you notify us now if you or your institution is planning to press release the article. All press must be co-ordinated with PLOS.

Thank you again for supporting Open Access publishing; we are looking forward to publishing your work in PLOS Computational Biology.

Best regards,

Vladimir B Teif, Ph.D.

Academic Editor

PLOS Computational Biology

Shaun Mahony

Section Editor

PLOS Computational Biology

# Editor report: Acceptance letter

**DOI:** 10.1371/journal.pcbi.1014557.r005

**Authors:** Vladimir Teif, Shaun Mahony

PCOMPBIOL-D-26-00605R1

Deciphering chromatin architecture and dynamics in Plasmodium falciparum using the nucDetective pipeline

Dear Dr Längst,

I am pleased to inform you that your manuscript has been formally accepted for publication in PLOS Computational Biology. Your manuscript is now with our production department and you will be notified of the publication date in due course.

The corresponding author will soon be receiving a typeset proof for review, to ensure errors have not been introduced during production. Please review the PDF proof of your manuscript carefully, as this is the last chance to correct any errors. Please note that major changes, or those which affect the scientific understanding of the work, will likely cause delays to the publication date of your manuscript.

Soon after your final files are uploaded, unless you have opted out, the early version of your manuscript will be published online. The date of the early version will be your article's publication date. The final article will be published to the same URL, and all versions of the paper will be accessible to readers.

For Research, Software, and Methods articles, you will receive an invoice from PLOS for your publication fee after your manuscript has reached the completed accept phase. If you receive an email requesting payment before acceptance or for any other service, this may be a phishing scheme. Learn how to identify phishing emails and protect your accounts at https://explore.plos.org/phishing.

Thank you again for supporting PLOS Computational Biology and open-access publishing. We are looking forward to publishing your work!

With kind regards,

Kannan R K Kuppusamy, B.TECH BIOTECHNOLOGY

PLOS Computational Biology | Carlyle House, Carlyle Road, Cambridge CB4 3DN | United Kingdom ploscompbiol@plos.org | Phone +44 (0) 1223-442824 | ploscompbiol.org | @PLOSCompBiol
